# A Physically Constrained Deep-Learning Fusion Method for Estimating Surface NO_2_ Concentration from Satellite and Ground Monitors

**DOI:** 10.1021/acs.est.4c07341

**Published:** 2024-11-20

**Authors:** Jia Xing, Bok H. Baek, Siwei Li, Chi-Tsan Wang, Ge Song, Siqi Ma, Shuxin Zheng, Chang Liu, Daniel Tong, Jung-Hun Woo, Tie-Yan Liu, Joshua S. Fu

**Affiliations:** † Center for Spatial Information Science and Systems, George Mason University, Fairfax, Virginia 22030, United States; ‡ Department of Civil and Environmental Engineering, The University of Tennessee, Knoxville, Tennessee 37996, United States; § Hubei Key Laboratory of Quantitative Remote Sensing of Land and Atmosphere, School of Remote Sensing and Information Engineering, Wuhan University, Wuhan, Hubei 430000, China; ∥ Microsoft Research AI for Science, Beijing 100080, China; ⊥ Graduate School of Environmental Studies, Seoul National University, Seoul 08826, Korea

**Keywords:** TROPOMI satellite, NO_2_, physically constrained, deep learning, model-measurement fusion

## Abstract

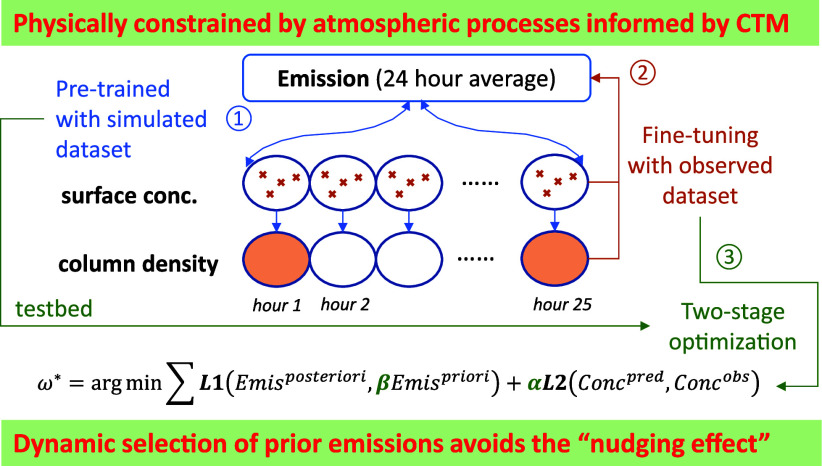

Accurate estimation of atmospheric chemical concentrations from multiple observations is crucial for assessing the health effects of air pollution. However, existing methods are limited by imbalanced samples from observations. Here, we introduce a novel deep-learning model-measurement fusion method (DeepMMF) constrained by physical laws inferred from a chemical transport model (CTM) to estimate NO_2_ concentrations over the Continental United States (CONUS). By pretraining with spatiotemporally complete CTM simulations, fine-tuning with satellite and ground measurements, and employing a novel optimization strategy for selecting proper prior emission, DeepMMF delivers improved NO_2_ estimates, showing greater consistency and daily variation alignment with observations (with NMB reduced from −0.3 to −0.1 compared to original CTM simulations). More importantly, DeepMMF effectively addressed the sample imbalance issue that causes overestimation (by over 100%) of downwind or rural concentrations in other methods. It achieves a higher *R*^2^ of 0.98 and a lower RMSE of 1.45 ppb compared to surface NO_2_ observations, overperforming other approaches, which show *R*^2^ values of 0.4–0.7 and RMSEs of 3–6 ppb. The method also offers a synergistic advantage by adjusting corresponding emissions, in agreement with changes (−10% to −20%) reported in the NEI between 2019 and 2020. Our results demonstrate the great potential of DeepMMF in data fusion to better support air pollution exposure estimation and forecasting.

## Abstract 2

This study introduces a novel physically constrained deep-learning fusion method for accurately estimating the atmospheric surface concentration to improve better exposure estimates for health assessment of air pollution.

## 1 Introduction

Atmospheric chemicals play a crucial role in air quality, climate, and ecosystems. Fully understanding their spatiotemporal variation is essential for assessing their impacts on human health^1^ and climate change^2^ and for supporting effective control strategies. In recent years, an increasing number of observations have become available,^3^ ranging from enhanced ground-based measurements for real-time in situ concentration monitoring to the deployment of advanced satellites for providing extensive global coverage. Additionally, advanced methods for estimating atmospheric chemical concentrations from satellite and ground monitors coupled with numerical model simulations have been continuously developed, particularly driven by the growth of machine learning techniques.^4^ However, challenges still exist in accurately estimating the surface concentration due to spatial and temporal discontinuities in observations and limitations in the data fusion methods used to interpolate data from multiple sources. Specifically, ground monitors are densely located in urban areas, leading to a sample imbalance in the spatial distribution between training and prediction data sets, which hampers the accuracy of spatial interpolation using traditional machine learning methods.^5^ Additionally, satellite measurements capture specific conditions only at certain cloud-free overpassing times of the day, making it challenging to fill in data for those missing hours.^6^ This issue is particularly problematic for species with strong diurnal variations, such as NO_2_.^7^ Additionally, machine learning models, often seen as black boxes, can artificially adjust concentration fields without considering physical realities, such as increasing the concentration levels in rural areas with limited emission sources.^8^ It is important to make an accurate adjustment of corresponding emissions to match with the fused concentration, following the physical connections as in an assimilation study.^9^ Therefore, a numerical model based on physical laws, like the chemistry transport model (CTM), is crucial to provide a robust scientific basis for reasonably fusing the limited observations. Some machine learning studies incorporate numerical simulation as an additional feature for training, enabling the interpolation of observations.^10^ Alternatively, some studies estimate surface concentrations directly from satellite measurements based on the numerical model-simulated column-to-surface ratio.^11−13^ Both approaches leverage the advantage of numerical simulations to maintain spatiotemporal continuity and capture spatial gradients, assuming the accuracy of baseline concentration estimations. However, most of the time, the accuracy of these simulations is often compromised in regions with limited access to crucial input data, such as high-quality emission data. Updating emissions periodically is labor-intensive and time-consuming,^14^ hindering real-time monitoring and adaptation. Such limitations also exist in traditional numerical model-based assimilation methods,^15,16^ such as the Kalman filter and Four-Dimensional Data Assimilation (FDDA), which face additional computational challenges and difficulties in accounting for uncertainties from various data sources. Advanced machine-learning methods have significant potential to enhance the fusion of multisource data sets from various observations and numerical model simulations, while developing effective strategies for data fusion that wisely integrate these different types of observations is crucial.

Given the spatiotemporally limited observed data sets, model simulations offer a significant advantage in creating data sets for training, making them ideal for data-driven methods such as machine learning. Using numerical model simulations to train and develop machine learning models provides a good physical constraint based on physical laws and can serve as a testbed to fully evaluate the model’s ability,^5^ especially considering that observations are often too limited to represent the entire space. As demonstrated in our previous study (i.e., DeepSAT4D^6^), leveraging numerical simulations to establish the correlation between column density and surface concentration and then applying real satellite observations successfully estimates detailed concentrations across the entire vertical profile; however, ground measurements have not been considered and may suffer from uncertainties in the satellite data or numerical model. Previous deep-learning-based inverse modeling, such as those using a deep-learning-based surrogate chemistry transport model (DeepCTM) for autogradient adjustment of emissions,^17^ or a Bayesian variational autoencoder (VAE),^8,18^ can efficiently adjust emissions. However, these methods rely solely on ground-level concentrations, neglecting the integration of diverse data sources and suffering from spatiotemporal sample-imbalance problems.

As a follow-up of our previous VAE inverse modeling study,^8^ here, we propose a novel, deep learning model-measurement fusion method (noted as DeepMMF) by using emissions as a constraint for atmospheric chemical concentrations to address the limitations of current fusion techniques, particularly the sample-imbalance problem. Specifically, we first establish the correlations among emissions, meteorology, and concentrations, as inferred from numerical models that adhere to physical laws. This step provides a pretrained basis for better representing these relationships, leveraging the more abundant data set available from numerical models compared to observational data. Subsequently, we use the observational data set to fine-tune the pretrained model, adjusting the concentration estimates to align with actual measurements. The emission will be simultaneously updated to such a corresponding adjustment of concentration to ensure adherence to physical laws, as if high concentrations are observed in a particular location, and they can be traced to either local emissions or external sources transported into the area, following atmospheric dynamics like diffusion and advection, driven by meteorological conditions. We also use numerical model simulation data sets as a testbed to evaluate and optimize the selection strategy for the determination of hyperparameters associated with the new optimized VAE model. By incorporating emission constraints into the loss function during model training (eq 1), we avoid unrealistic emission adjustments, such as unwarranted increases in areas without new emission sources (e.g., rural areas). This study applies it to the NO_2_ species over the Continental United States (CONUS) domain with a 12 km × 12 km spatial resolution at the daily average level. Noting that though the work presented here is only suitable for the nationwide exposure analysis for it has a relatively coarse resolution, regional exposure, and emissions, it can easily be applied to other pollutants, regions, and hourly resolutions with the corresponding data sets, particularly applied at a finer (1 km) spatial resolution, which is more suitable for assessing city-level health impacts.

## 2 Method

### 2.1 The Framework of DeepMMF

The principle of the DeepMMF is to effectively incorporate multiple data sets by leveraging their advantages and mitigating their limitations. The numerical model like CTM provides a better representation of atmospheric physical processes, including emissions, diffusion, advection, and deposition and provides an abundance of data for training. This makes it ideal for pretraining the machine-learning model as a surrogate for the numerical model, as demonstrated by DeepCTM in this study. We rely on the correlations inferred by the CTM, which excels at representing the relationships between emissions and concentrations under specific meteorological conditions rather than on its baseline concentration predictions. However, discrepancies between CTM outputs and reality must be addressed by fine-tuning with ground and satellite observations. Although these observations have limited spatial and temporal coverage, they provide accurate, real-world measurements that are crucial for calibration.

**Figure 1:**
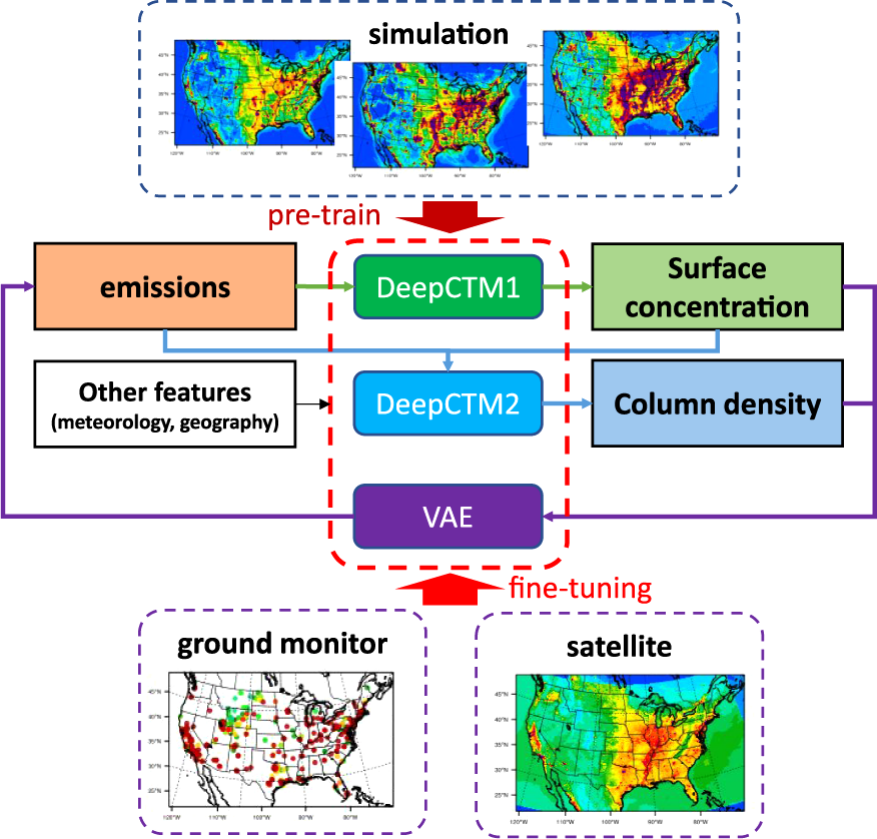
Framework of the proposed physically constrained deep-learning data fusion (DeepMMF) method based on the VAE.

As illustrated in Figure 1, the surrogate model is first trained using abundant simulation data to mimic the CTM and establish relationships among emissions, concentrations, and meteorological conditions. Two DeepCTM models (forward models, following the same input/output variables as the numerical model, detailed in Figure S1) are established to provide real-time predictions of the surface concentration and column density, respectively, inferred from CTMs but with significantly improved computational efficiency. The replacement of traditional CTMs by DeepCTM is crucial because the following VAE optimization requires real-time calculation of the loss function, which considers both emission changes and concentration updates. These models use the same emissions and meteorological variables as inputs and serve as decoders for VAE training. The DeepCTM2 model, which predicts column density, will also leverage surface concentration from DeepCTM1 as an input feature, as the NO_2_ column density is more challenging to estimate than surface NO_2_ due to the complex vertical distribution of NO_2_ in the atmosphere. By incorporating surface NO_2_ and total NO_*x*_ emissions, the model gains valuable information to better capture the vertical NO_2_ profile, leading to more accurate predictions of the NO_2_ column density. Consequently, it relies on the output from the DeepCTM1 model, which predicts the surface concentration, during the VAE training process. The encoder in VAE (backward model, emission will replace the concentration as the output, detailed in Figure S2) is also pretrained using multiple simulation data sets with various combinations of emission and meteorological conditions. This approach allows it to capture a wide range of variability, serving as an inversion modeling method for estimating emissions from inputs of both the surface concentration and column density. Such abundant simulation data provide good constraints following the physical laws. After pretraining of VAE, these simulations will be replaced by surface measurements and satellite observations, which have either limited spatial coverage or temporal frequency. Eventually, the fine-tuned DeepMMF model generates assimilated concentrations by fusing simulation data with multiple observations and then estimates the corresponding emissions adjusted to match the fused concentrations. It should be noted that the inversion process may suffer from uncertainties stemming from the CTM, the observations, and the machine-learning model itself. Therefore, any discrepancy between baseline emissions and posterior emissions does not necessarily imply an error in prior emissions. Nevertheless, the changes in the posterior emissions between two years predicted by the DeepMMF will be helpful for updating the year-to-year emissions in an efficient way, by only taking the change ratio, which is mostly driven by the variation of observations.

### 2.2 Data Set

The simulation data were derived by running the Weather Research and Forecasting (WRF)^19^ model and the Community Multiscale Air Quality (CMAQ)^20^ at a 12 km × 12 km spatial resolution with up to 35 vertical layers using the same configuration as our previous study.^21^ We conducted a base run for the year 2019 (noted as “Baseline”) using the U.S. EPA National Emissions Inventory (NEI) emission inventory of 2019.^22^ To adequately capture the range of emissions and meteorological variations, three additional hypothetical scenarios were conducted: one with zero emissions (noted as “Hypo-1”), one with double the base emissions (“Hypo-2”), and one with updated meteorology for 2020 (noted as “Hypo-3”). At the time of our study, emissions data for 2020 were unavailable. Directly using CMAQ simulations to interpolate missing temporal and spatial data for 2020 was also not feasible due to significant emission changes during the COVID-19 pandemic. This delay in emissions development often hinders real-time updates of concentration fusions. Although the 2020 NEI emissions data became available in December 2023,^23^ we did not incorporate it into our numerical CTM modeling to maintain consistency with real-time conditions, as our goal is to provide near-real-time (NRT) fusion where emissions cannot be frequently updated. Instead, we used the latest NEI emissions data from 2019 and 2020 from the U.S. EPA to compare with the top-down posterior emission estimates from the DeepMMF model. Another challenge is that using the same baseline prior emissions to estimate emissions for two different years with significant changes (such as during COVID-19 from 2019 to 2020) can underestimate the differences, as both years will be nudged to the same emission level. To address this issue, we propose a new two-stage strategy leveraging dynamic prior emissions.

The DeepMMF hyperparameter (ω*) loss function is optimized as follows:
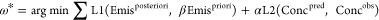
1

where L1 represents the first part of the loss, which is the divergence of posterior emissions from the prior emissions. L2 represents the second part of the loss, which is the divergence of the predicted surface concentration and column density from the observations. The parameter α represents the weighting loss between L1 and L2. The parameter β represents the dynamic level of prior emissions relative to the baseline emissions (e.g., 2019 NEI in this study).

In the first stage, we use the same base prior emissions to determine the optimized weighting loss between emissions and observations (α) by sampling values over a wide range (0.1 to 100) and selecting the turning point as the optimized value. Given that there are two observation data sets, the weighting loss from surface concentration and column density is determined by their respective variances according to our previous study,^8^ with the sum of weighting losses from surface concentration and column density considered as the total observation loss. In the second stage, we select the optimized weighting loss coefficient but with different prior emission levels (β), such as ranging from 0 (no emissions) to 2 (double emissions). We chose the case with the least observation loss as the optimized prior emissions. This design allows the model to select different levels of prior emissions for each year, avoiding the nudging effect on their differences. Additionally, we used the numerical simulation data as a testbed to illustrate the nudging problem and validate the proposed strategy. By mimicking the training and testing process with the selected simulation data in grid cells corresponding to ground monitor sites and time steps corresponding to satellite overpassing times, we utilized the “ground-truth” emission data and full spatiotemporal coverage concentrations for validation. This testbed approach aids in validation and refining of the training strategy during the two-stay optimization process (detailed in Text S1 and Figure S3).

The satellite-observed NO_2_ column density was obtained from the Tropospheric Monitoring Instrument (TROPOMI)^24^ product, which has a local pass time of around 14:00 each day, thus lacking information for the 23 h between measurements. The TROPOMI NO_2_ data are filtered by its quality flag, as defined as “qa_value” by the Algorithm Theoretical Basis Document (ATBD), by a value of 0.50. As described by the ATBD, a qa_value of over 0.50 represents that the NO_2_ column data are sufficiently good for comparisons against models or column observations (including vertical profiles) and include data for special situations (snow/ice or cloudy scenes). Ground measurements were obtained from the US EPA Air Quality System (AQS), which includes approximately 400–500 sites that measure NO_2_, excluding 77 near-road sites. These sites were aggregated into around 300 12 × 12 km grid cells. When multiple AQS sites fell within the same grid cell, their measurements were averaged (which occurred in about 5% of the cases). They only represent a very small percentage (<1%) of the entire CONUS domain, which comprises 117 130 grid cells (265 rows × 442 columns). In addition, AQS sites are primarily located in urban areas with heavy sources of pollution, leading to significant sampling imbalance. Training a model solely based on observation data can be insufficient because false causalities can develop with a small sample size. To address the limitation, we only apply the observation data during the fine-tuning process after the DeepMMF pretraining with the simulation data set.

### 2.3 Training

The training of the two DeepCTM models follows the same methodology as our previous study,^6^ incorporating both forward and backward directions to account for satellite measurement times around 14:00 local time, using the ConvLSTM model structure.^25^ The models were trained using data from the first 25 days of each month for one year (300 days/year) and tested on the remaining days. Predictions for the 24 h time series are initiated from local time 14:00 on the previous day. For data augmentation, we introduced random cropping of the feature maps to dimensions of 60 rows x 60 columns. During training, we utilized the mean squared error (MSE) loss function over a total of 3000 epochs. This number of epochs proved sufficient for achieving good performance in both the training and testing phases. Our learning rate started at 0.0001 and linearly decayed to zero by the end of the training process. We employed the Adam optimizer^26^ to enhance model convergence.

The DeepCTM models effectively capture spatial and temporal variations with acceptable performance for both surface concentration (DeepCTM1: *R*^2^ > 0.9, |NMB| < 0.05 in training and *R*^2^ > 0.8, |NMB| < 0.25 in testing) and column density (DeepCTM2: *R*^2^ > 0.85, |NMB| < 0.1 in training and *R*^2^ > 0.8, |NMB| < 0.20 in testing), as shown in Figures S4–S5 and S6–S7, respectively.

For the VAE pretraining using simulation data, the trained DeepCTM models act as the decoder to train the encoder using the UNet-LSTM framework.^17^ The loss function is carefully designed not only to consider the discrepancy between the adjusted emissions and the prior emissions but also to include the discrepancy between the DeepCTM1-predicted surface concentration using β-adjusted emissions and that using prior emissions but also the discrepancy between the DeepCTM2-predicted column density using β-adjusted emissions and that using prior emissions. This ensures that the DeepMMF model will not simply memorize the emission patterns, which can be quite similar each day in prior emissions for each scenario. This design is similar to the traditional VAE structure with direct surface-level observation training, as used in our previous study.^8^ The trained model successfully reproduces emission variations under different scenarios (“Baseline” and “Hypo-2”) with acceptable performance (*R*^2^ > 0.9, |NMB| < 0.15), as presented in Figure S8.

During fine-tuning, ground measurement and satellite observation data replace the simulated surface concentration and column density to account for the loss from the discrepancy between their predictions with β-adjusted emissions. Additionally, we extend the constraint on emissions from total emissions used in pretraining to sectoral emissions. This ensures that the β-adjusted emissions follow sectoral patterns and align more closely with reality, although the same weighting for each sector is simply applied in this study. It should be noted that these constraints have limitations, particularly for wildfires, which may differ significantly from prior emissions. Furthermore, uncertainties in wildfire emissions are extremely large, even in prior emissions.

## 3 Results and Discussion

### 3.1 Fused Concentration

**Figure 2:**
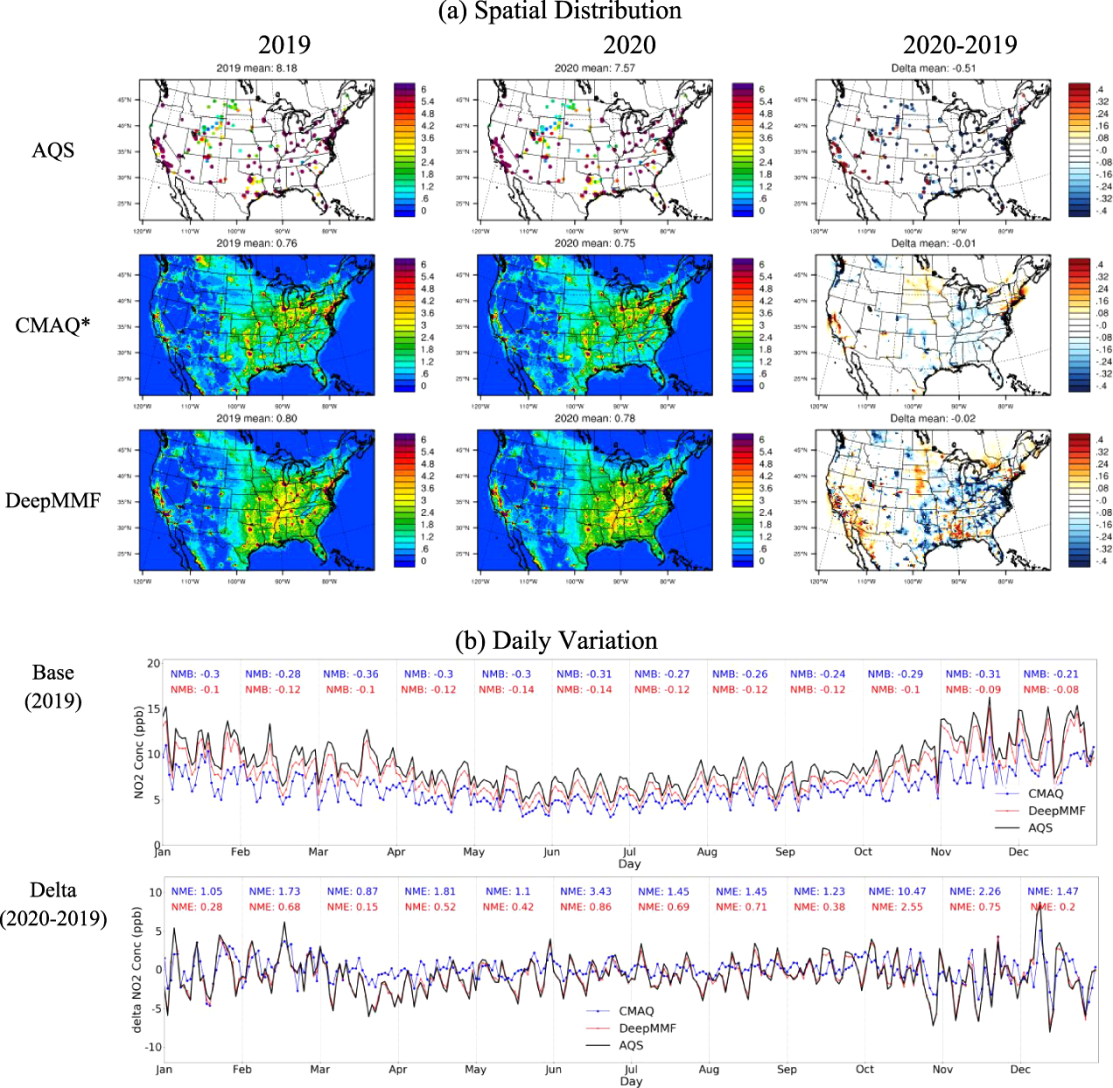
Comparison of the observed, simulated, and fused NO_2_ surface concentration (unit: ppb, *the CMAQ runs for 2019 and 2020 are using the same emissions, as “Baseline” and “Hypo-3”).

In general, the surface concentrations predicted by DeepMMF exhibit spatial patterns consistent with those from the original CMAQ model. However, DeepMMF shows higher concentrations of NO_2_, fused by AQS measurements, than those simulated by CMAQ (Figure 2). This suggests that the original CMAQ may underestimate surface NO_2_ concentrations,^27^ with the largest low biases (NMB over −0.36) in March 2020 during the COVID-19 period, as the reduction of anthropogenic emissions stemming from the shutdown was not considered in the prior emissions used for simulations. The negative biases were reduced by DeepMMF across the year, and also, there were no systemically low biases in March (NMB = −0.1, which is at the same level as other months from −0.08 to −0.14), implying to be more consistent toward the AQS observations, and DeepMMF has well captured the reduction of emission during the COVID-19 period. It does not exactly match with AQS, constrained by the discrepancy from the prior emissions, considering the uncertainties from the model itself, also the systematic errors from the comparison stemming from the factors including coarse model spatial resolutions,^28^ and the CMAQ model mechanism such as the uncounted canopy effects^29^ from plant or building structures, which may also contribute to the biases in simulating gaseous species like NO_2_.

Additionally, the significant reduction observed in AQS measurements in the eastern US is also reflected in DeepMMF, where the reduction is more pronounced than that in the original CMAQ runs with the same prior emissions but different meteorological conditions. It is evident that the change in emissions between 2019 and 2020 is the dominant factor driving the change in surface NO_2_ concentration, though meteorological variations also contribute slightly to this change. The DeepMMF successfully reflected the day-to-day variation in surface NO_2_, and it also captured the reduction during the COVID period in March 2020, with NME reduced from 0.87 to 0.15.

Satellite observations also contribute to the differences in DeepMMF-predicted NO_2_ column density compared to the original CMAQ simulations (Figure S9). The fusion with satellite data leads more consistent column density estimated by DeepMMF toward the satellite than the original CMAQ, demonstrated by the slighted decrease of |NMB| from <0.33 in CMAQ to <0.18 in DeepMMF. With large underestimation in CMAQ from March to September, besides the uncertainties in emissions, such biases might be related to missing emission sources such as lightning and aircraft, or downwelling of stratospheric NO_*y*_ produced from N_2_O near tropopause^30^ where a regional model like CMAQ fails to capture such extra increase. The constraints by other factors like prior emission and ground measurement in DeepMMF prevent DeepMMF from artificially adjusting toward the satellite observations with a suspicious increase of NO_*x*_ emission to compensate the low biases, which are not mainly driven by the ground emissions. The incorporation of satellite data in DeepMMF also results in a more pronounced reduction in column density than that seen in the original CMAQ in the southeastern US and changes the trend from an increase to a decrease in the northeastern US. Consistent with AQS measurements, the changes in column density observed by the satellite indicate reductions in emissions between 2019 and 2020.

The DeepMMF is still able to capture well the day-to-day variation of the NO_2_ column density observed by the satellite, while it suffers significant underestimation from March to September, ensuring its ability to estimate the emission changes from the fusion with observations. Such results suggest that DeepMMF successfully integrates information from both AQS and satellite measurements into the original CMAQ, providing a more accurate representation of the spatiotemporal pattern of surface NO_2_ concentrations.

### 3.2 Emission Adjustment

**Figure 3:**
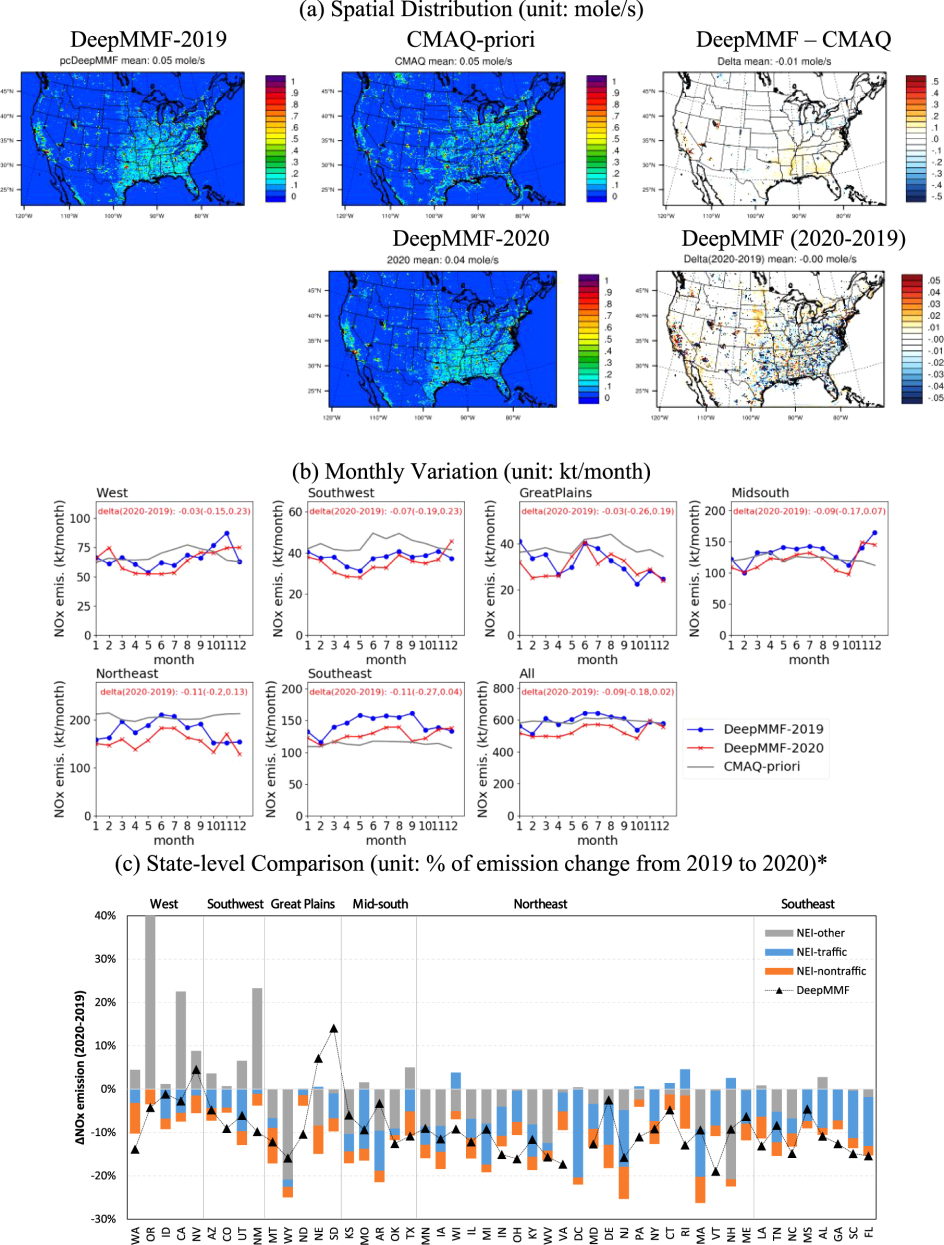
Comparison of prior NEI emission changes (2020–2019) used in CMAQ and posterior emission adjusted by DeepMMF (*the DeepMMF predicted DC is 205%, which is not shown in the figure).

The advantage of DeepMMF lies in its fused concentration, which is naturally correlated with changes in emissions interacting with meteorological factors rather than artificially increasing concentrations without any constraints on emissions. To further evaluate the performance of DeepMMF in estimating top-down emissions using the inverse method, we compared the prior emissions used in the original CMAQ simulation to the posterior emissions adjusted by DeepMMF to match the fused observations. As shown in Figure 3, the results indicate higher emissions in the southeastern US, which is expected because the fused concentration in DeepMMF is also enhanced, primarily driven by satellite observations. The NO_2_ column density simulated with CMAQ tends to be substantially underestimated compared to satellite observations in the southeastern US (Figure S9a). Conversely, lower emissions are observed in the northern US due to the lower satellite-observed column density compared to that simulated with prior emissions in CMAQ. The DeepMMF also captures changes in emissions from 2019 to 2020, showing reductions mostly in the eastern US due to the COVID-19 shutdown, with its influence lasting from March until September (up to 30%, as shown in Figure 3b), and increases in the western US due to wildfires. Emissions in the northeastern US are also reduced according to DeepMMF, despite satellite measurements indicating an increase from 2019 to 2020. This increase is mainly driven by meteorological conditions rather than emissions, as the increase ratio is even larger in the CMAQ simulation between 2019 and 2020 with the same emission levels (Figure S9a). These results demonstrate DeepMMF’s ability to separate the driving factors for changes in concentration, whether they stem from emissions or meteorological conditions.

We also compared the DeepMMF-adjusted top-down emissions with the bottom-up 2019 NEI from the U.S. EPA and estimated 2020 pandemic emissions using human activity information (Figure 3c).^31,32^ In general, DeepMMF reflects the overall increase or decrease patterns across the states. For instance, most states in the west and southwest regions show an expected increase in NO_*x*_ emissions driven by no traffic-related (NEI-other) sources (e.g., wildfire activities), which largely offset the reductions driven by traffic sectors. The DeepMMF exhibits either an increase or decrease in NO_*x*_ emissions depending on the net effects from nontraffic and traffic factors; however, uncertainties, particularly from wildfire emission sources, may also contribute to biases in the bottom-up emissions in NEI. On the other hand, most states in the northeast and southeast regions exhibit strong reductions in NO_*x*_ emissions. The DeepMMF effectively captures this reduction, exhibiting a comparable decreasing ratio of around −10% to −20%. However, a suspiciously large increase ratio was found in DC by DeepMMF, likely due to the smaller baseline emission (see Figure S11), whereas NEI shows a significant decrease ratio. This discrepancy likely arises from uncertainties in the observations and spatial resolution, as the changes of NO_*x*_ emissions are mainly contributed by on-road traffic in DC, which requires much higher resolution to observe than satellite or AQS measurements, which are usually away from highways. Thus, further improvement using a high-density observation network with ultrafine downscaling modeling is necessary to improve performance and achieve consistent estimations between top-down and bottom-up methods.

### 3.3 Sensitivity to the Prior Emissions Selections

The testbed analysis underscores the importance of dynamically selecting prior emissions for accurately estimating emission changes (Text S1 and Figure S3). Directly using the 2019 NEI as a prior emission for another year, such as 2020, may not be suitable. To address this, we conducted a sensitivity analysis by comparing the results of DeepMMF using fixed prior emissions versus dynamic β-adjusted emissions during the fine-tuning process. The selection of prior emissions is detailed in Text S2 and Figure S10.

We compared the differences between using fixed prior emissions (based on the baseline emission) for both years and dynamic prior emissions (i.e., baseline for 2019 and a smaller emission level, 0.8 times the baseline, for 2020). The results suggest that using fixed priors results in smaller changes in emissions compared with dynamic priors in most states (Figure S11). Clearly, dynamic prior emissions are crucial for accurately estimating changes in emissions; otherwise, changes in emissions will be significantly underestimated. Our proposed two-stage strategy allows for more flexibility in applying the model in years where prior emissions data may not be available, enhancing its usefulness.

### 3.4 Interpretation of DeepMMF in Estimating Emissions from Various Features

**Figure 4:**
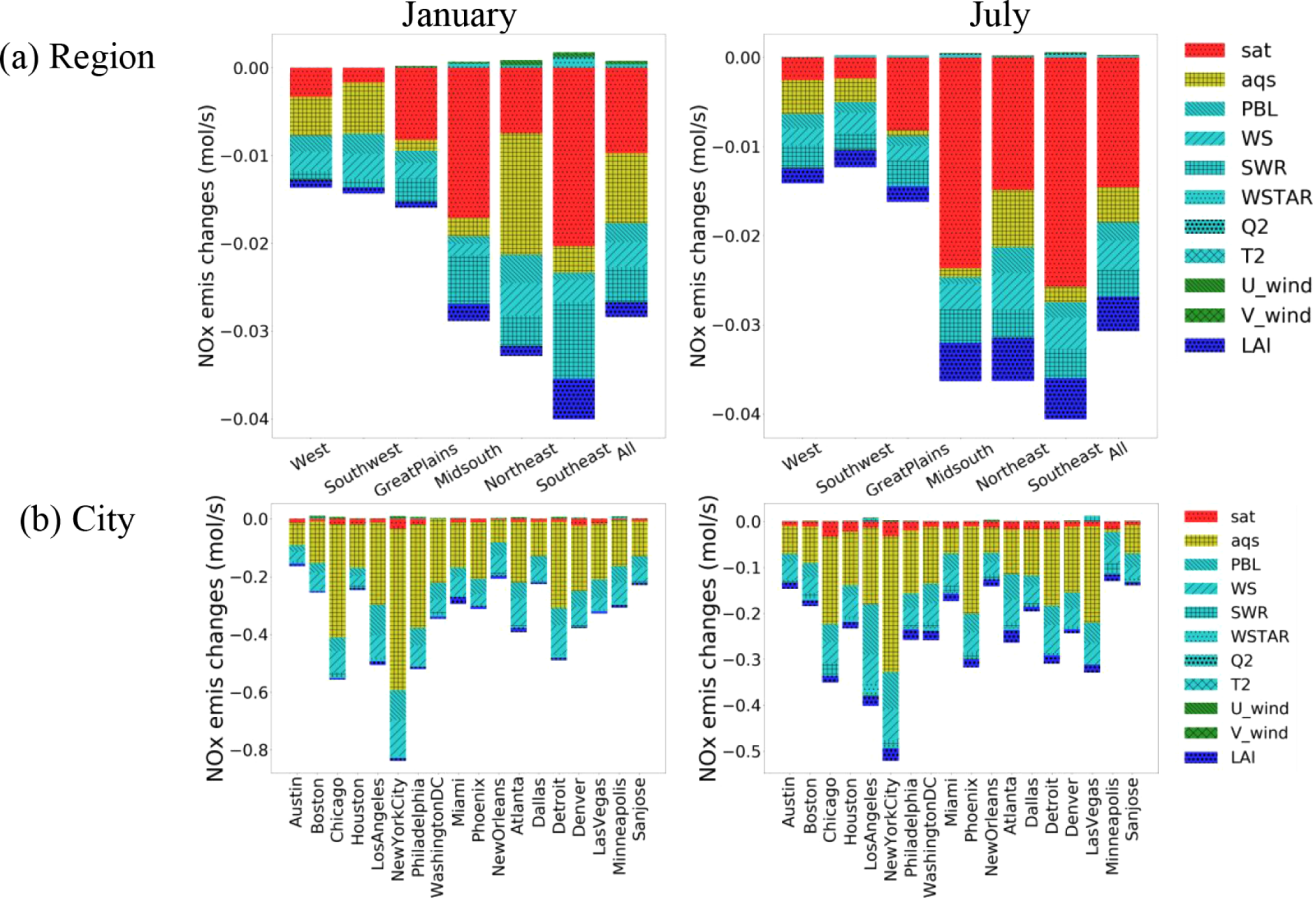
Sensitivity analysis for the importance in features (monthly average).

Although the DeepMMF machine learning model lacks transparency in its data handling compared to a physical model, it is still possible to investigate its underlying calculations. This can be achieved through sensitivity analysis by individually modulating the input and observing the model’s predictive responses. Following the same strategy as in our previous studies,^33^ we reduced the input features by 20% (e.g., decreasing ground T by 2 degrees) and regarded the difference from the base case as the contribution to the feature, as shown in Figure 4, which illustrates this correlation between emissions and concentrations under different meteorological conditions. For instance, the lower measured NO_2_ levels indicate smaller emissions, which is consistent with our expectations, and these correlations are also affected by meteorological variables, particularly the planetary boundary layer height (PBL), wind speed (WS), and short-wave radiation (SWR); their reduction leads to a reduced estimation of emissions. This is because smaller PBL and WS imply relatively stable atmospheric dispersion conditions and lower SWR implies relatively weak atmospheric oxidation capacity (oxidize NO_2_ to nitrate acid, acting as a loss), thus requiring smaller emission sources to maintain the same concentration levels. Therefore, the estimated emissions will be smaller. Such insights into the DeepMMF response to changes in individual features demonstrate its reasonability in dealing with the correlations between the inputs and output, adhering to the physical laws, as expected.

The results demonstrate the significant contribution from the satellite observations (noted as “sat” in Figure 4) and the AQS data set, accounting for more than more than 50% of the total response.

Another interesting finding is that satellite observations play a more important role on a regional scale, particularly in areas such as the Great Plains, Midsouth, and Southeast, where ground measurements are limited. However, they are less important in regions with dense AQS coverage, such as the Northeast, Southwest, and at city grid cells (proximate to the city area to represent the urban environment), influenced by the different weighting between ground measurements (with 24 hourly records per day) and satellite columns (with 1 h record per day). The DeepMMF model successfully balances the weighting and role of multiple observations.

### 3.5 Comparison of Fused Concentrations Among Different Methods

One of the advantages of DeepMMF is its ability to address the limitations of traditional fusion methods, which often suffer from a limited observation data set that either faces sample imbalance problems or is not efficiently fused with observations. We compared the results of fused concentrations obtained by using different methods.

The first two methods are traditional machine learning models based on either decision trees (LightGBM)^8^ or deep neural networks (ResNet).^5^ These models aim to establish correlations between column and surface concentrations using features, such as meteorological and geographical variables. However, they suffer from sample imbalance problems, as most ground measurements are located in urban areas with high pollution levels. This imbalance can lead to overestimations in downwind and rural areas, even when neighborhood features or additional samples from the simulation data are added to the training set.

Another method is the machine learning-based column-surface ratio method (DeepSAT4D),^6^ which uses the column-surface ratio simulated by a numerical model to estimate surface concentration from column density. This method has advantages over traditional column-surface ratio methods because it does not require additional CTM simulations, having already been trained with deep learning. However, it heavily depends on the accuracy of the numerical model and does not utilize ground-based measurements from AQS.

**Figure 5:**
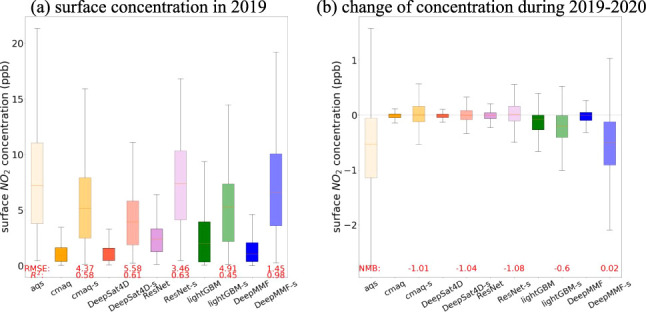
Comparison of fused concentration and changes using different methods and settings (the model with “-s” represents only selecting the grids that match with the AQS sites; the statistics for each model are provided at the bottom, highlighted in red).

In contrast, DeepMMF addresses these issues more effectively, providing a more accurate and robust fusion of observations and model simulations. As presented in Figure 5, DeepMMF excels in effectively fusing concentration data from both the satellite column and AQS ground measurements, capturing variations in concentration between years, with a larger *R*^2^ of 0.98 and a smaller RMSE of 1.45 ppb than others with an *R*^2^ of 0.4–0.7 and an RMSE of 3–6 ppb. The grid-to-grid scatter plot comparing the annual mean levels of surface NO_2_ across AQS sites is provided in Figure S13, and multiple AQS observations within a single grid cell (approximately 5% of the total cases) are averaged into a single value. Without AQS fusion, DeepSAT4D predictions significantly underestimate concentrations, closely resembling the original CMAQ simulation. Conversely, the sample imbalance problem causes significant overestimations in both LightGBM and ResNet predictions (see Figure S12), although their predictions at monitor sites are closer to AQS measurements than DeepSAT4D. The DeepMMF successfully constrains its predictions to align with the original CMAQ (without suspicious large increases on a regional scale like other methods) while remaining consistent with AQS measurements at monitor sites, demonstrating a successful fusion result. Additionally, the DeepMMF captures the changes during 2019–2020 in AQS concentrations much better than other methods, as the |NMB| in DeepMMF is 0.02, which is smaller than other models over 0.6.

### 3.6 Implication and Future Work

In summary, the DeepMMF model exhibits excellent performance in fusing multiple data sets from different sources, including simulations and various observational data with different temporal and spatial coverage. It also provides an insightful example of effectively coupling a machine learning model with a physical model through physically constrained machine learning. Machine learning, as a data-driven method, requires an abundant data set for training to better capture nonlinear systems like atmospheric chemistry. Physical models have the advantage of generating data under various conditions, which is crucial for better training machine learning models. Unlike most previous studies that directly feed physical model simulation data into the machine learning model structure as a feature or input, the physical constraints on the machine learning defined in this study involve integrating the simulated data into the training process during pretraining. This approach can effectively avoid uncertainties in the physical model itself while maintaining the machine learning model as an efficient, CTM-free model for the applications. Another advantage of using physical models to assist machine learning is their role as a testbed, which can efficiently validate and improve the machine learning model. This study demonstrates this benefit through a two-stage optimization strategy and the selection of prior emissions and its ground-to-column ratio in one typical city (taking Austin as an example, all 18 cities can be found in Figure S14).

**Figure 6:**
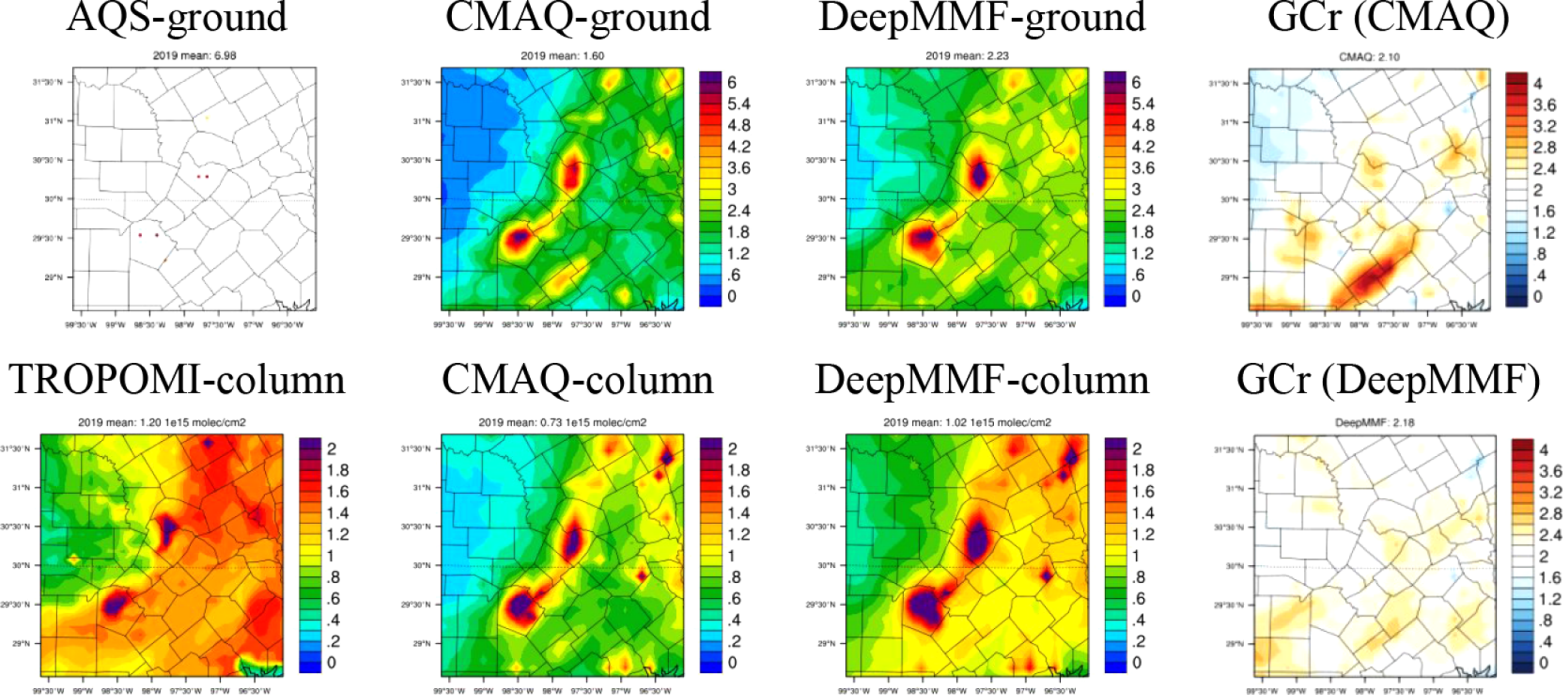
Comparison of simulated, observed, and fused ground and column density of NO_2_.

While this study focuses on a national scale, leveraging the availability of CMAQ simulations at a 12 km resolution, we strongly recommend applying this method at the 1 km urban scale in future studies. Doing so would enhance human exposure assessments and reduce uncertainties stemming from sample imbalance. Data fusion at finer scales is particularly prone to these uncertainties, given the steep emission gradients (e.g., diffusion from sources), more complex meteorological conditions, urban canopy effects, and fewer ground measurements for training. These factors make it even more challenging for sparse ground measurements to represent broader spatial patterns accurately, as seen in Figure 6; the AQS sites are sparse and the spatial gradient is seeable even at the 12 km resolution in 18 major US cities, though the DeepMMF can keep similar spatial pattern as original CMAQ for both ground and column density (and the ground-to-column ratio, noted as GCr), implying its ability in dealing with the urban-rural differences in vertical profiles, as high ground-level concentrations are typically indicative of denser urban emissions; future applications could benefit from higher-resolution CMAQ simulations,^28^ supported by increased observations, such as hourly satellite data (e.g., TEMPO^34^) and ground-level measurements from low-cost sensors. While training the machine learning model with higher resolution requires a large memory resource, the DeepCTM we designed previously for the vertical profile of NO_2_ has difficulty being applyied in this study, which has much higher spatial/vertical resolutions and limited the accuracy particularly for applying the averaging kernel to better calculate the NO_2_ column due to the different sensitivity of the satellite signal to each vertical layer. While considering that we mainly constrain the NO_2_ for rural based on the satellite but the urban is mostly constrained by the ground measurement, the uncertainties might be not that important but it should be considered in future if the computational resources are enough to support the full vertical structure of NO_2_ prediction for this study.

This enhancement could also improve its ability to adjust emissions by sector-level. In this study, emissions were constrained with the same weight ratio for each sector despite potential differences in uncertainties among sectors (e.g., point sources versus wildfires). Future developments could incorporate uncertainties in emissions based on factors such as emission factors and activity information used in prior emission calculations. Besides, optimizing column and surface weighting, considering these uncertainties, is crucial for accurately quantifying emissions. The uncertainty of satellite retrievals can be assessed through technical reports on remote sensing algorithms used for satellite signal retrievals. Ground measurements typically have smaller uncertainties due to high-accuracy equipment but may suffer from representativeness issues within a modeling grid cell, particularly in areas with heterogeneous emission distributions (e.g., near large point sources or roadsides with higher concentrations than downwind areas). Careful design and balance of weighting factors for each component, along with more abundant observations of high accuracy and additional spatial surrogate information, are necessary to enhance the reliability of the inversion study of DeepMMF in the future.

## Supporting Information Available

Detailed description about the two-stage strategy for the optimization of hyperparameter through Testbed and application (Texts S1 and S2); model structure of DeepCTM and encoder of VAE (Figures S1 and S2); the performance of DeepCTM1 and VAE (Figures S4–S8); NO_2_ column density (Figure S9); other model performance comparison (Figures S11 and S12); scatter plot for surface NO_2_ comparison (Figure S13); zoom-in US cities (Figure S14) (PDF)
