# Supplementary material for: A Physically Constrained Deep-Learning Fusion Method for Estimating Surface NO2 Concentration from Satellite and Ground Monitors
Source: Environ Sci Technol. 2024 Nov 20;58(48):21218–28. doi: 10.1021/acs.est.4c07341 (PMC11618989; doi:10.1021/acs.est.4c07341)
Supplement: Supplementary file 1 — es4c07341_si_001.pdf [file es4c07341_si_001.pdf]

Supplementary information for **A physically constrained deep-learning fusion method for estimating surface NO_2 concentration from satellite and ground monitors**

Jia Xing[1,2], Bok H. Baek[1,*], Siwei Li[3,*], Chi-Tsan Wang[1], Ge Song[3], Siqi Ma[1], Shuxin Zheng[4], Chang Liu[4], Daniel Tong[1], Jung-Hun Woo[5], Tie-Yan Liu[4], Joshua S. Fu[2]

[1] Center for Spatial Information Science and Systems, George Mason University, Fairfax, VA 22030, USA

[2] Department of Civil and Environmental Engineering, the University of Tennessee, Knoxville, TN 37996, USA

[3] Hubei Key Laboratory of Quantitative Remote Sensing of Land and Atmosphere, School of Remote Sensing and Information Engineering, Wuhan University, Hubei, 430000, China

[4] Microsoft Research AI for science, Beijing 100080, China

[5] Department of Environmental Planning, Seoul National University, Seoul, Korea

Correspondence to: Bok H. Baek (bbaek@gmu.edu); Siwei Li (email: siwei.li@whu.edu.cn)

Summary:

Pages: 23

Figures: S1-S14

Texts: S1-S2

Text S1 Two-stage strategy for optimization of hyperparameter through Testbed

As illustrated in our previous study[8], the weight of each loss component in the total loss function is a key parameter (α) in the DeepMMF that needs to be determined through optimization. This involves conducting multiple DeepMMF training sessions and testing the results to calculate both the loss of emissions and concentrations, ultimately identifying the optimal hyperparameter values. We introduce the simulation data as a testbed to design the optimization strategy for the selection of hyperparameters including the weight of each loss component and sensitivity to the prior emissions.

Dynamic selection of appropriate prior emissions and balancing the discrepancy between the divergence from prior emissions to posterior emissions and the divergence from observed concentration to fused concentration are crucial for solving the inversion problem in DeepMMF. As suggested in our previous study[5], using numerical model simulations as a testbed can be highly beneficial for providing "ground truth" to validate and improve machine-learning models. This method should be applied in various scenarios, including those established by numerical models, even though they might not fully reflect reality.

First, in Stage 1 we examine the method of using the same prior emissions for inversion. One scenario involves using lower prior emissions, where the concentration in a double-emission scenario ("Hypo-2") is used to retrieve the emissions, with the baseline emission as priori. The other scenario involves using higher prior emissions, where the concentration in a baseline emission scenario ("Baseline") is used to retrieve the emissions, but with double emissions as priori. We conducted multiple experiments with different weighting values (ranging from 0.1 to 100) for these two components in the loss function during training. Using the traditional L-curve method to select the optimal weighting value (α) for DeepMMF, results showed significant underestimation or overestimation (±20%) if using lower or higher prior emissions, respectively.

Their differences will also be considerably underestimated (the testbed suggested a change ratio of only 70%, while the ground truth is 100%).

In Stage 2, based on the optimized weighting value (β) (represented by two red points in Figure S3a), we conducted multiple experiments with different prior emissions ranging from nearly zero emissions (0.000001) to 2.4 times baseline emissions. Results indicate that the selection of prior emissions significantly impacts the performance in predicting concentrations. Interestingly, selecting double emissions as priori in Case 1 and selecting 0.9 times emissions as priori in Case 2 (green points in Figure S3b) exhibited considerably lower biases in predicting concentrations compared to the original cases (red points in Figure S3b). Both scenarios also achieved results closer to the ground truth, with the change ratio significantly reduced (estimated at 111%, closer to the ground-truth of 100%). Therefore, dynamically selecting prior emissions for estimating changes is crucial, particularly for this study when substantial changes are expected from 2019 to 2020 due to the COVID-19.

Text S2 Two-stage strategy for optimization of hyperparameter in 2019 and 2020

Following Stage 1, which involved optimizing the hyperparameters for weighting the two loss components, we conducted multiple experiments with different weighting values ranging from 0.1 to 100. The results indicate that all experiments lie on the L-curve, showing a decrease in the divergence from observed concentration to fused concentration as the divergence from prior emissions to posterior emissions increases. The optimal configuration, identified at the turn-around point, occurs with a weight of 0.7 (β) of the base prior emission, as indicated by the red points in Figure S10a. This suggests that further adjustments to the prior emissions provide only marginal benefits in reducing biases in concentration.

We applied the same weighting value (0.7) but with different prior emissions during VAE training in Stage 2, using various ratios of the 2019 NEI prior emissions, ranging from 0.7 to 1.4 times. The divergence from observed concentration to fused concentration in each case is compared in Figure S10b. For 2019, using the baseline emission as the prior emission results in the smallest discrepancy from the observation, which is also the optimized prior emission. In contrast, for 2020, using 0.8 times the baseline emission results in the smallest discrepancy from the observation, which is lower than the baseline emission, suggesting that a smaller prior emission should be used for 2020.

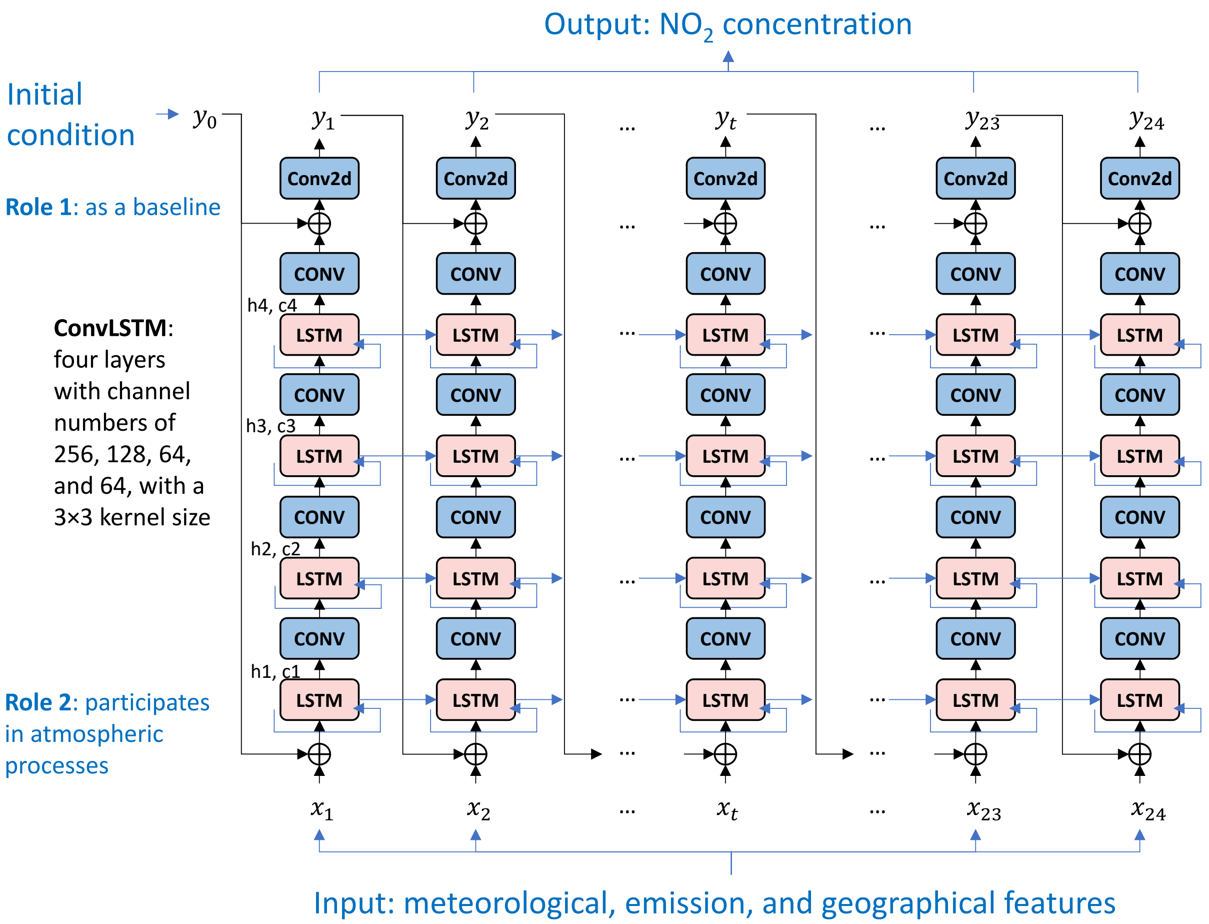

Figure S1. Model structure of DeepCTM, same as Li and Xing (2024), we use convLSTM model to predict the hourly concentrations with the time-series of meteorological factors observed over a 24-hour period, as well as time-independent geographical features, and daily emission data; In adherence to physical laws, we feed the initial condition into the model twice to account for its role in atmospheric physical and chemical processes and its function as a modulatable baseline to facilitate the training process. Furthermore, we've designed a dual-model that incorporates both forward and backward models concurrently, considering satellite measurements at 24-hour intervals, to constrain the concentration and mitigate the issue of error accumulation)

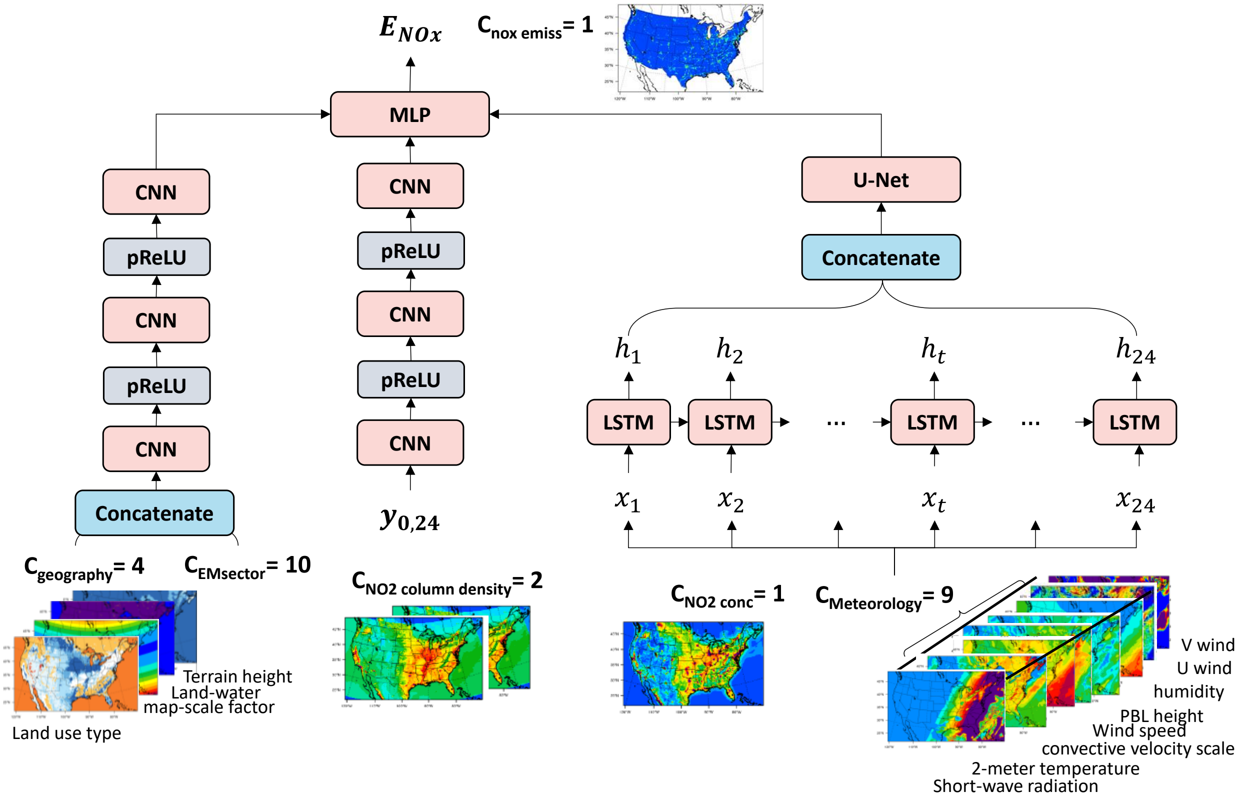

Figure S2. Model structure of encoder in VAE, same as Li and Xing (2024), we use UNet-LSTM to estimates emission adjustments to align with the observed concentration. We input all time-series features, including and ground-NO2 concentration and meteorological factors, into the LSTM layer to extract temporal features. These temporal features are then combined with time-independent features, such as terrain height and sectoral emission profiles (ratio to total emission), and the two-hour concentrations observed by satellites (at hour 0 and hour 24). This information is used to predict the 24-hour average emissions between these two time points

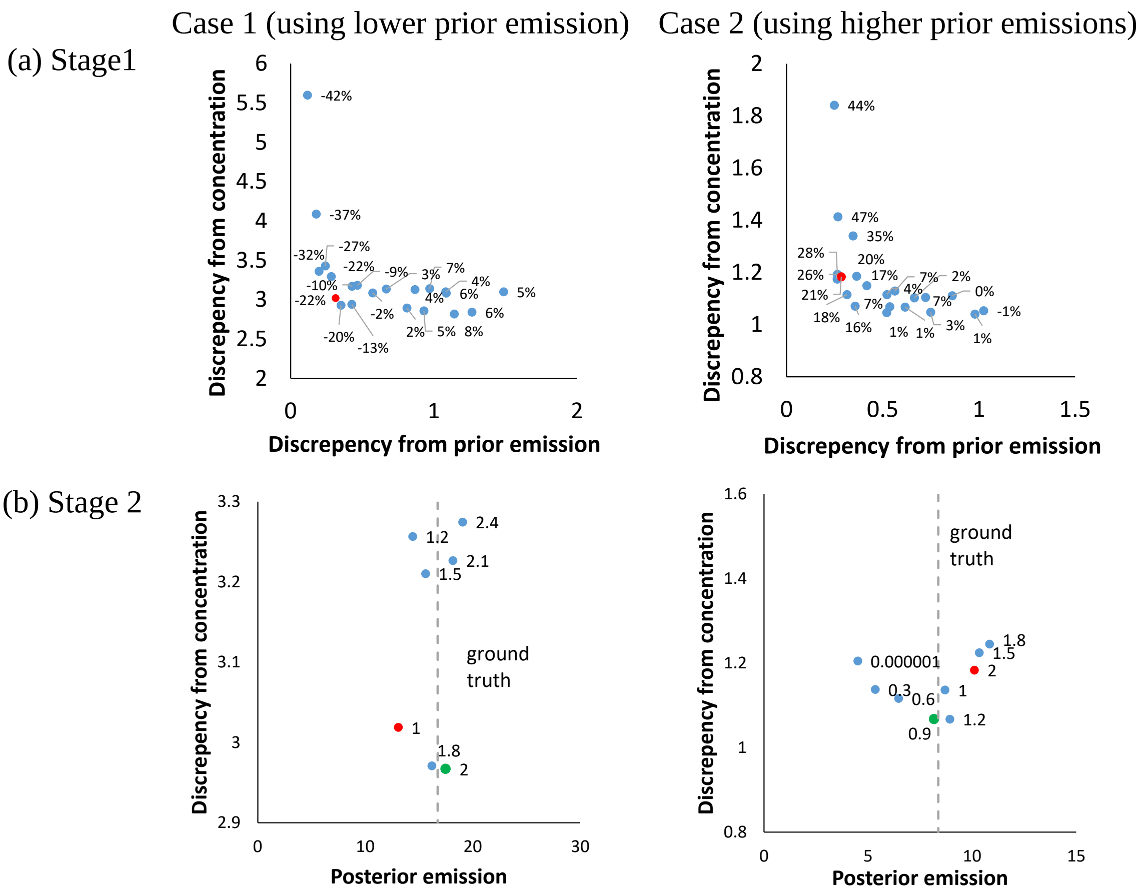

Figure S3. Testbed to validate the model performance in predicting the emissions by using different prior emissions (Stage 1 (a): the points are labeled by the normalized bias from the ground-truth; Stage 2 (b): the points are labeled by the prior emission ratio to the baseline emission)

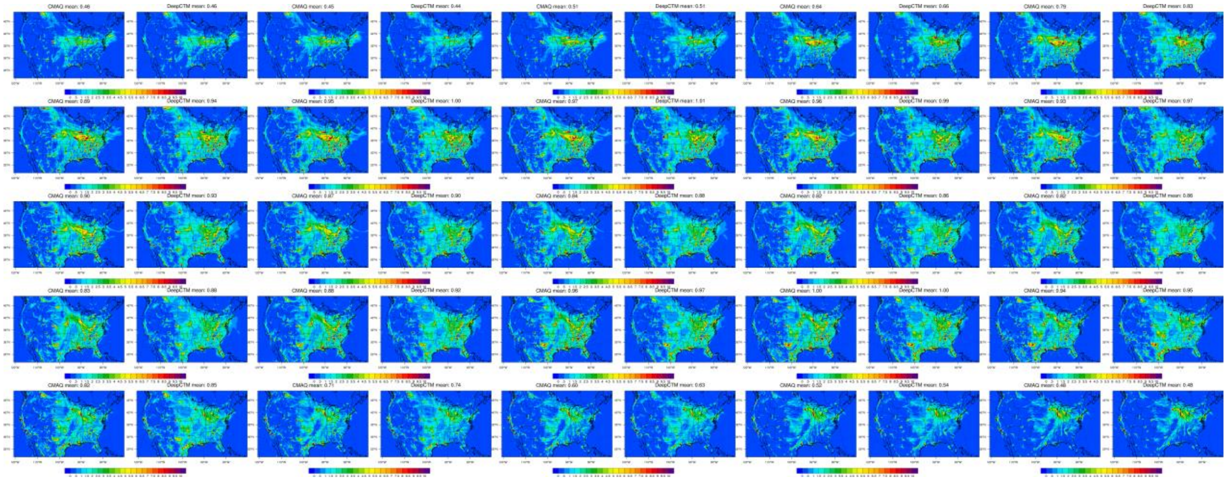

(a) spatial distribution

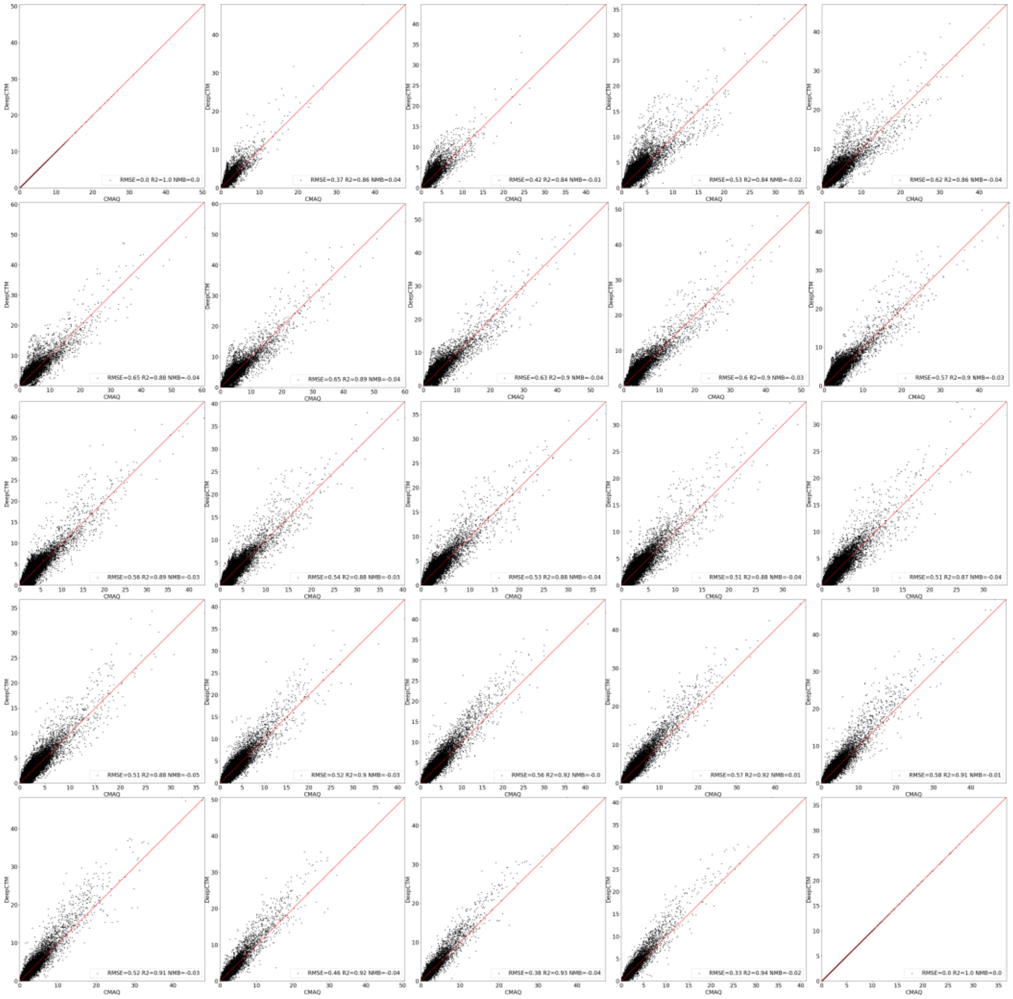

(b) point-to-point comparison

Figure S4. The performance of DeepCTM1 in estimating surface NO_2 concentration with emission and meteorology in a training example (January 6, 0 – 24 hour)

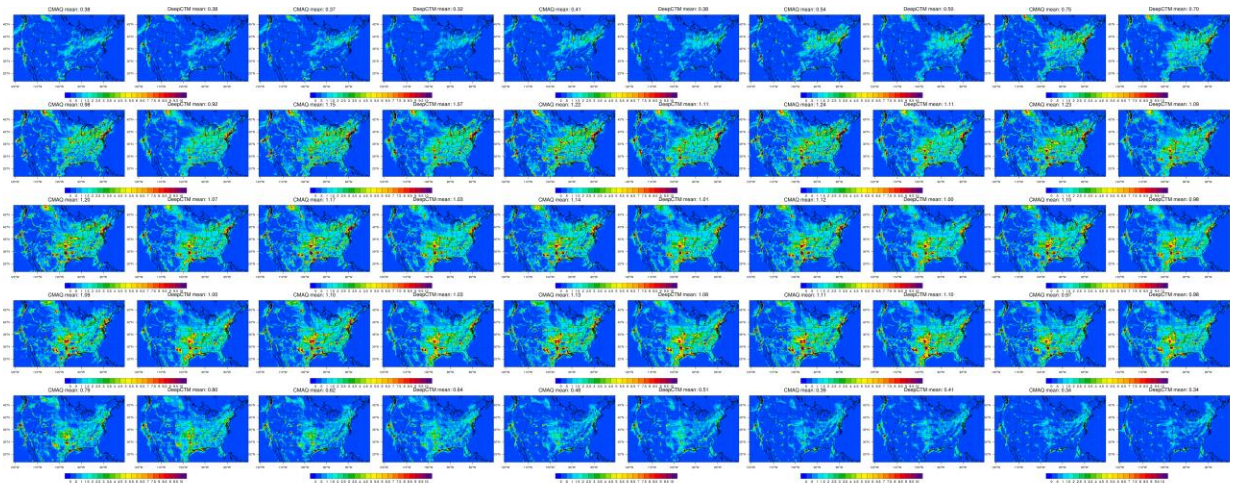

(a) spatial distribution

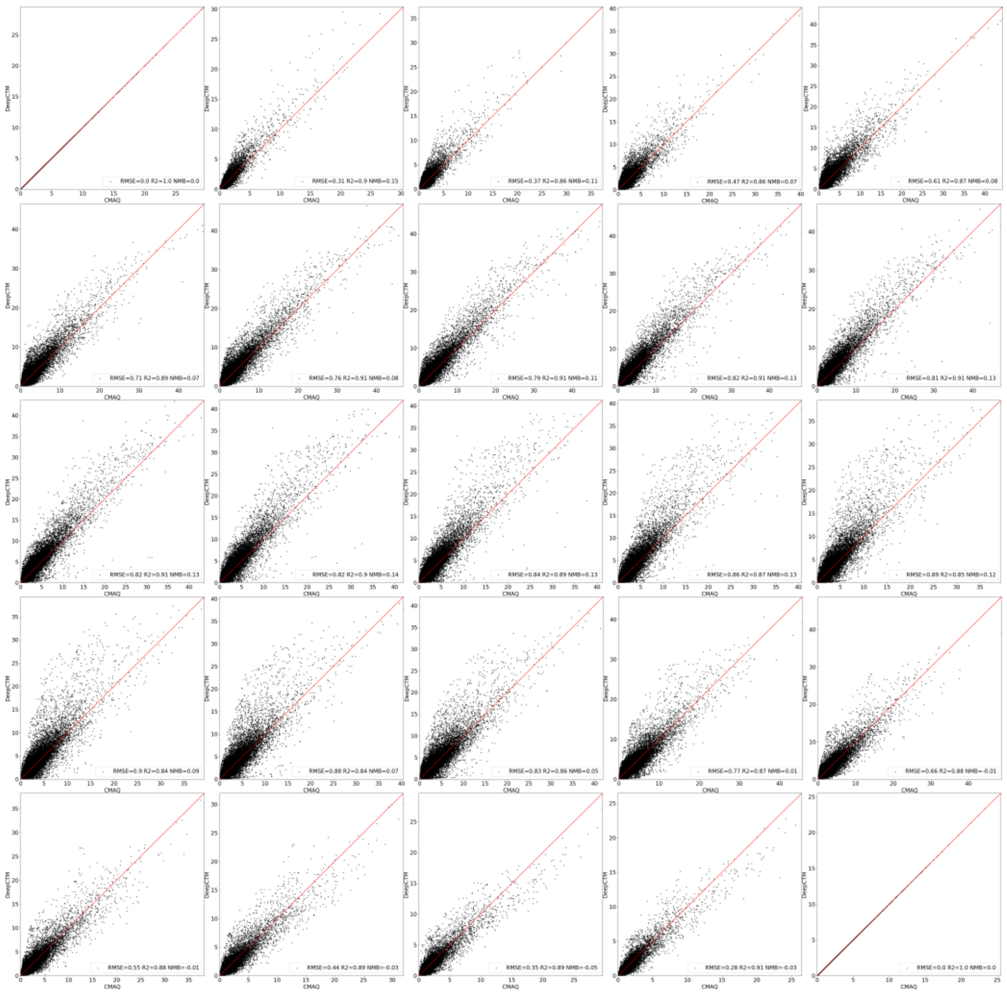

(b) point-to-point comparison

Figure S5. Same as Figure S1 but in a test example (January 26, 0 – 24 hour)

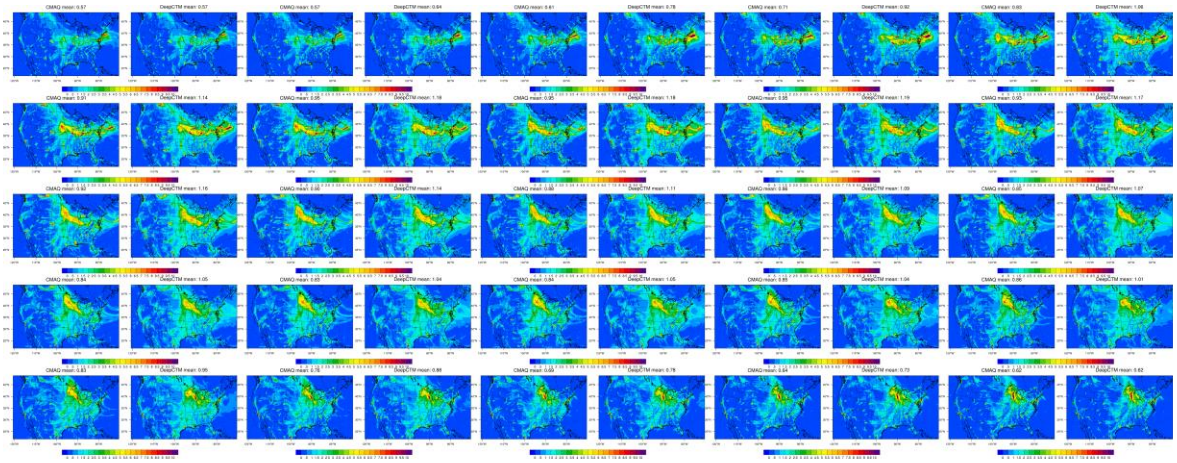

(a) spatial distribution

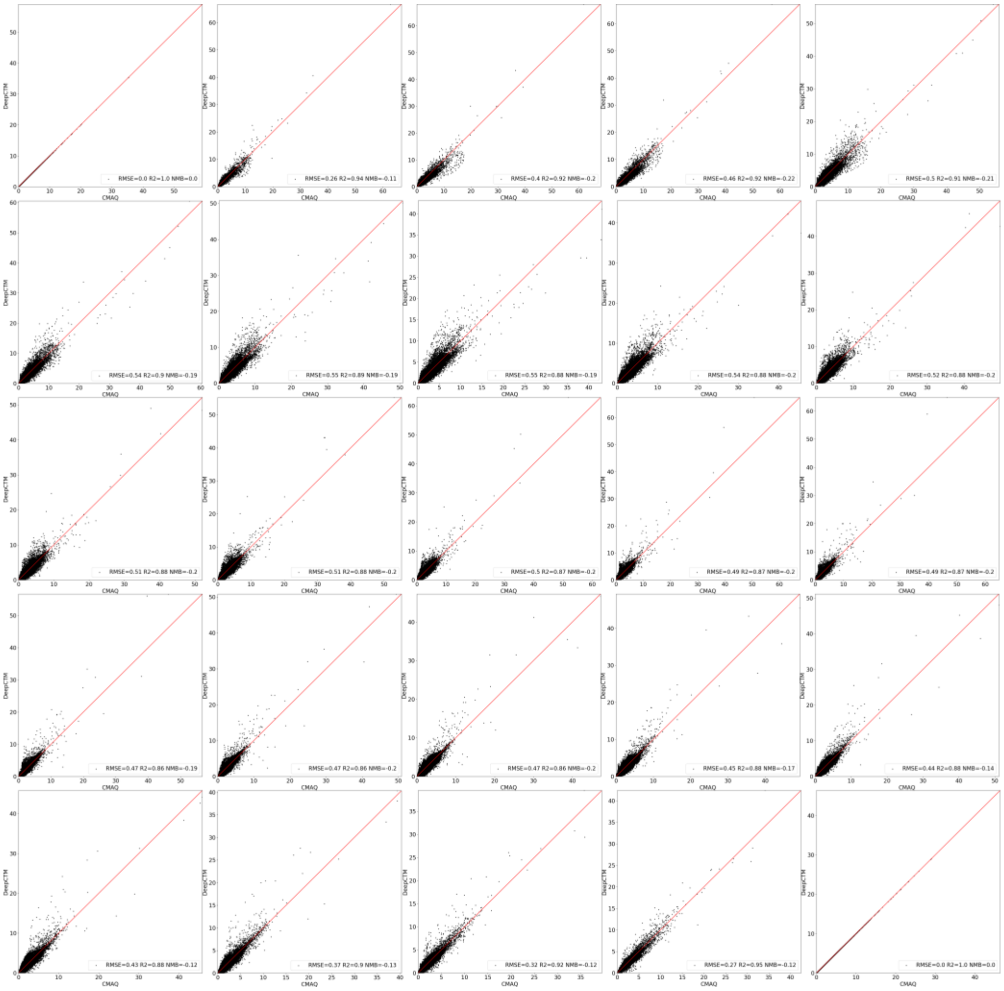

(b) point-to-point comparison

Figure S6. The performance of DeepCTM2 in estimating NO_2 column density with emission and meteorology in a training example (January 6, 0 – 24 hour)

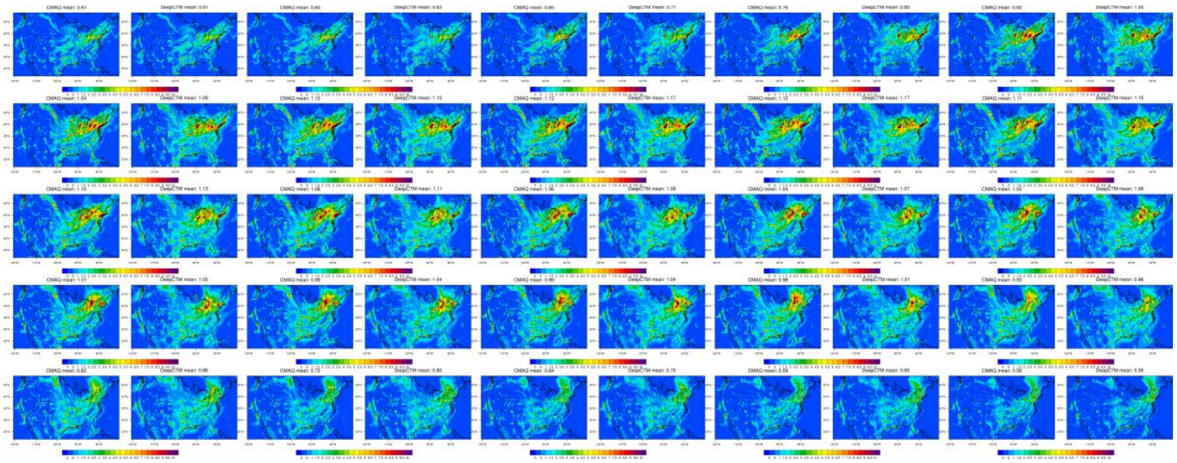

(a) spatial distribution

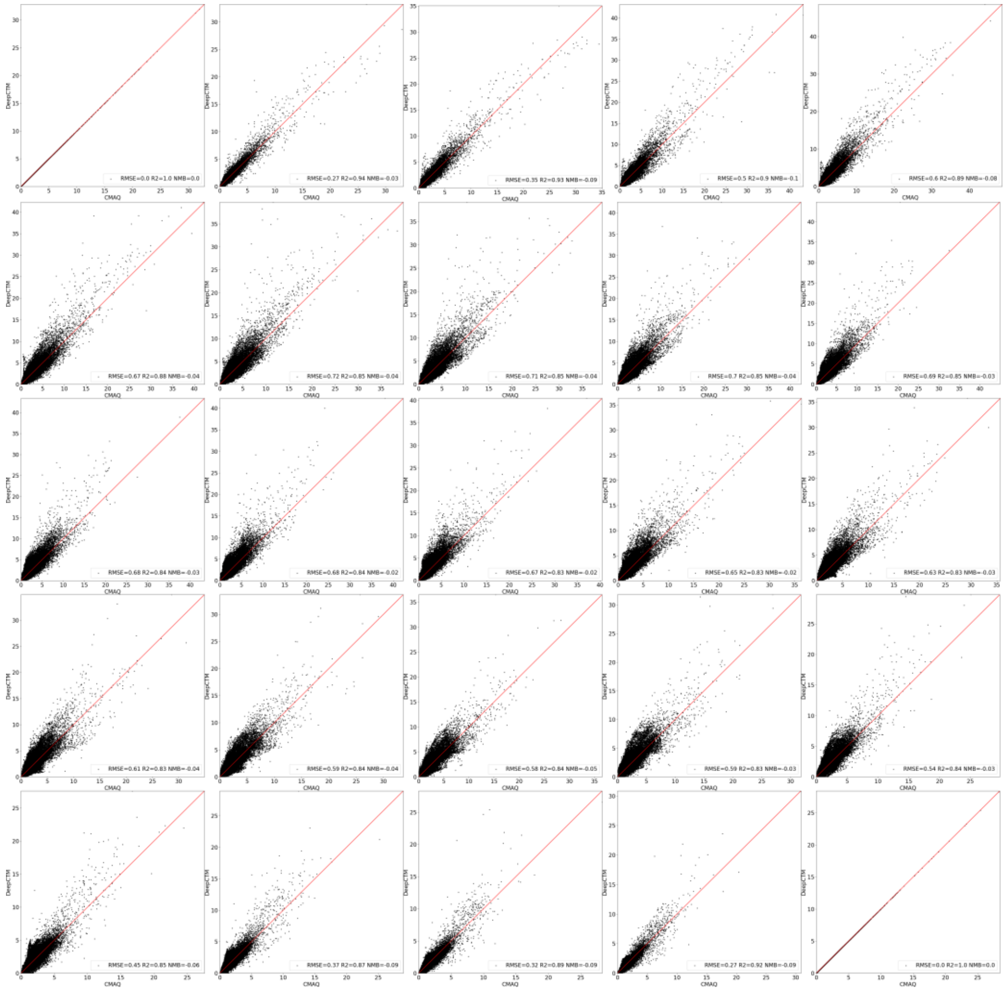

(b) point-to-point comparison

Figure S7. Same as Figure S3 but in a test example (January 26, 0 – 24 hour)

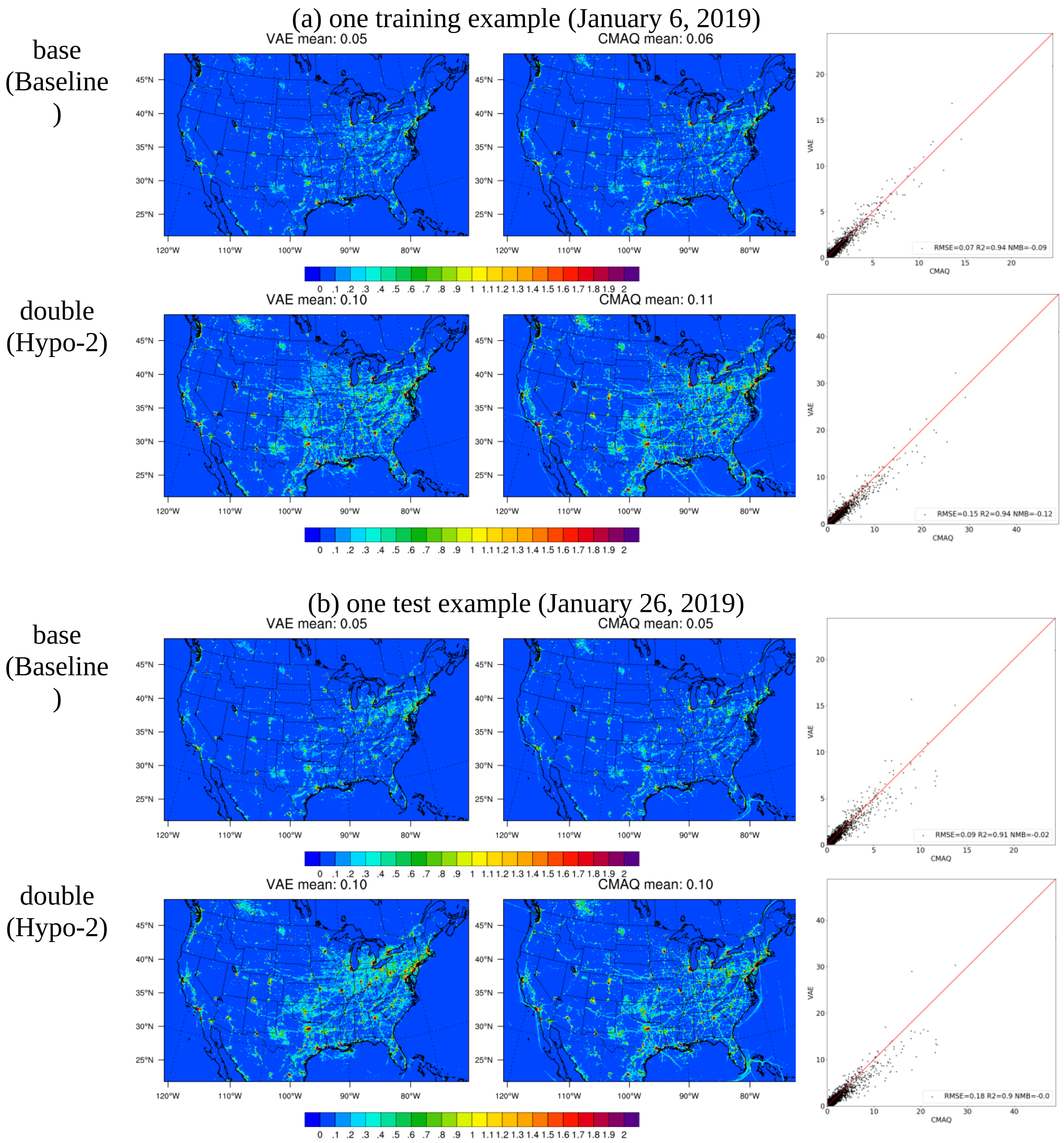

Figure S8. The performance of pre-trained VAE in estimating emissions with concentrations and meteorology in two scenarios

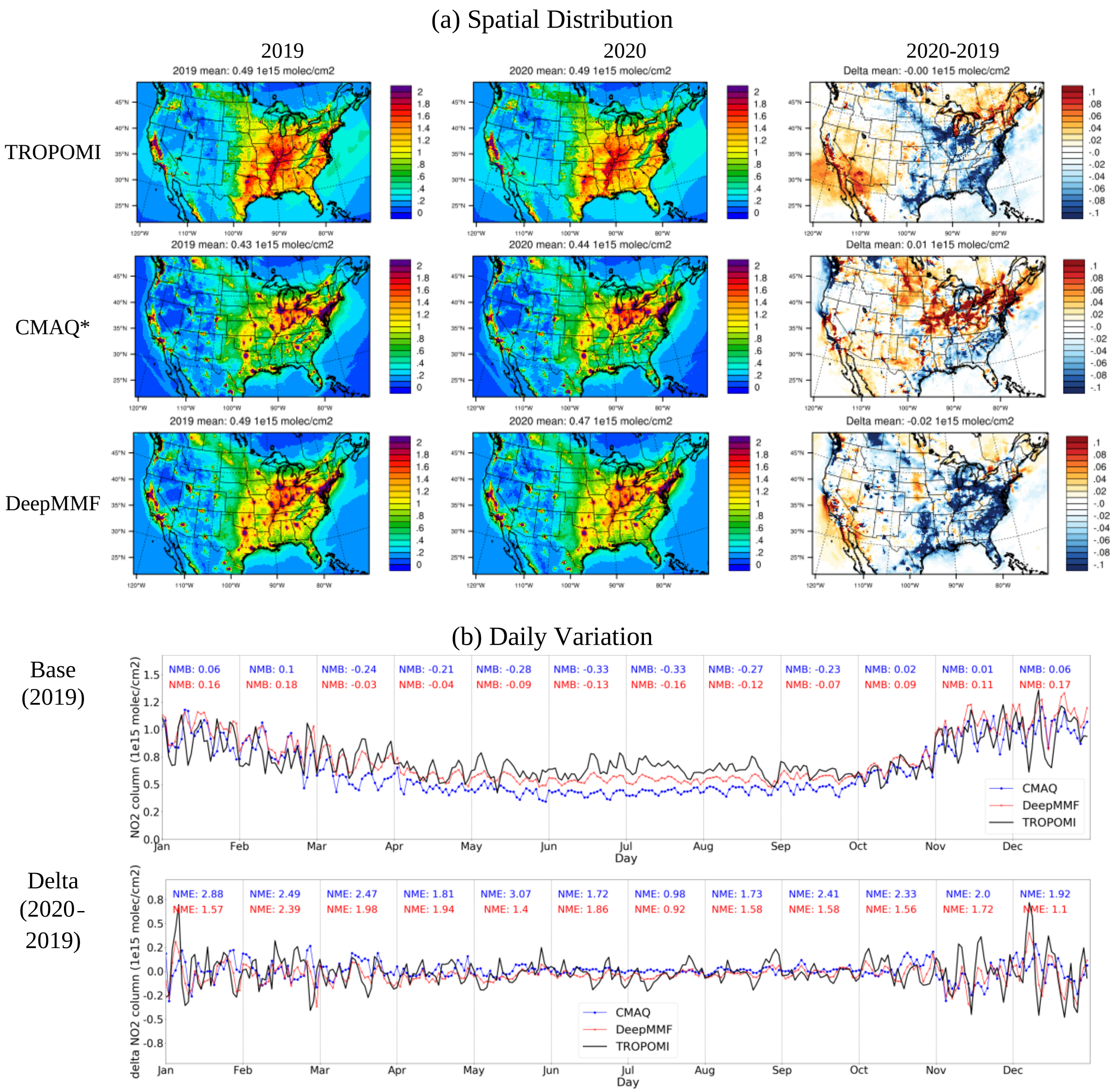

Figure S9. Comparison of simulated, observed, and fused NO_2 column density (*the CMAQ run for 2019 and 2020 are using the same emissions, as “Baseline” and “Hypo-3”)

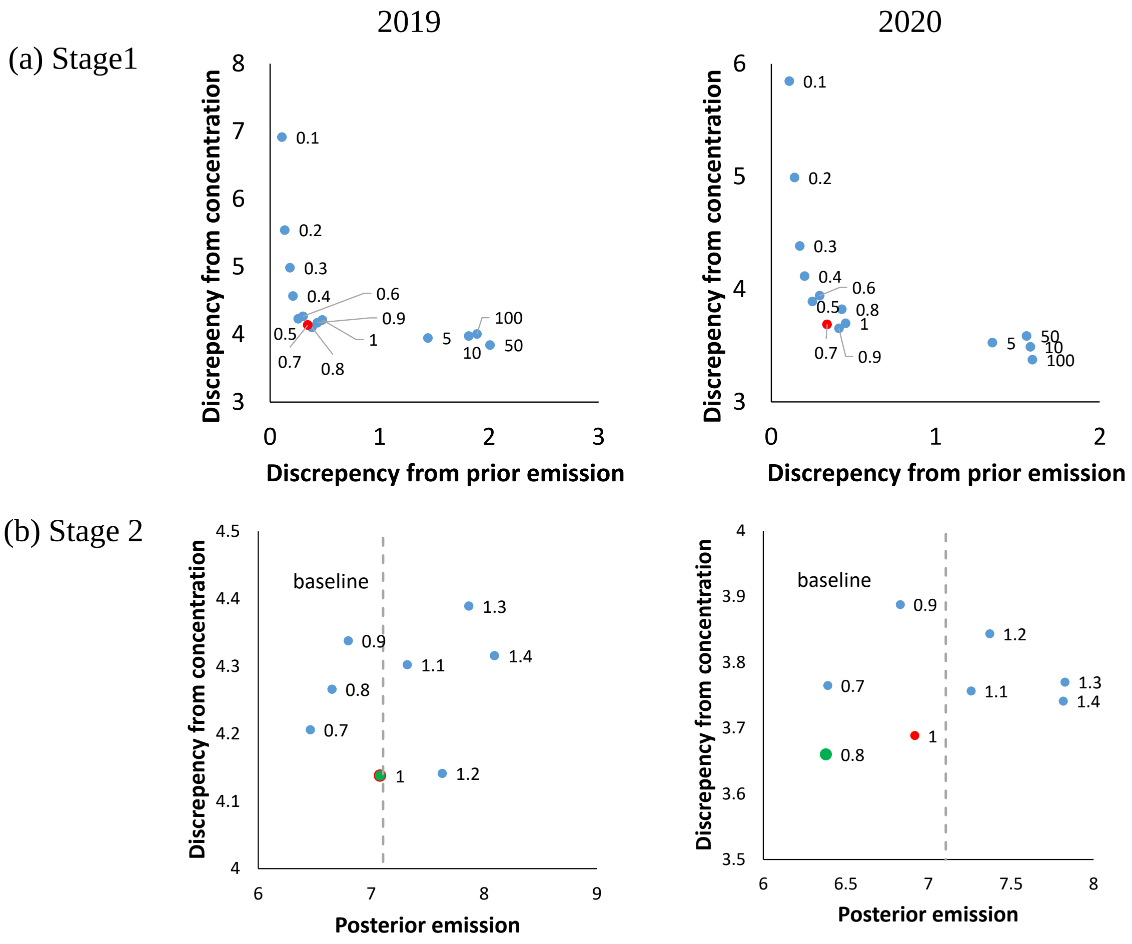

Figure S10. Correlation between the discrepancy in posterior and prior emissions and that in predicted concentrations of both surface and column density in 2019 and 2020 retrievals (a: the points was labeled by the weighting value; b: the points was labeled by the prior emission ratio to the baseline emission)

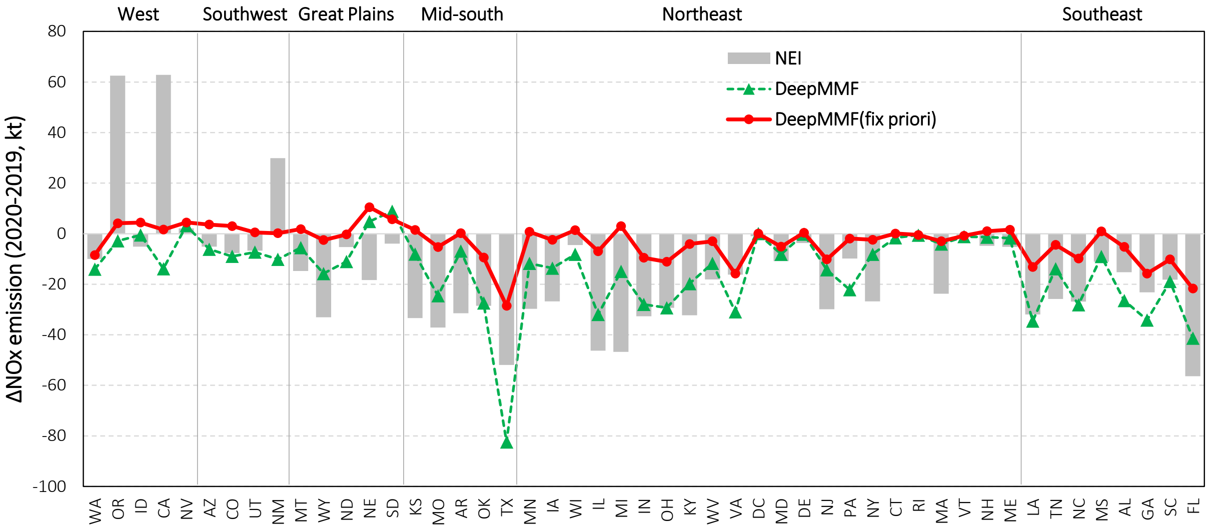

Figure S11. Comparison of the change changes in NEI inventory, retrieved by DeepMMF and DeepMMF with fix prior emissions across the states

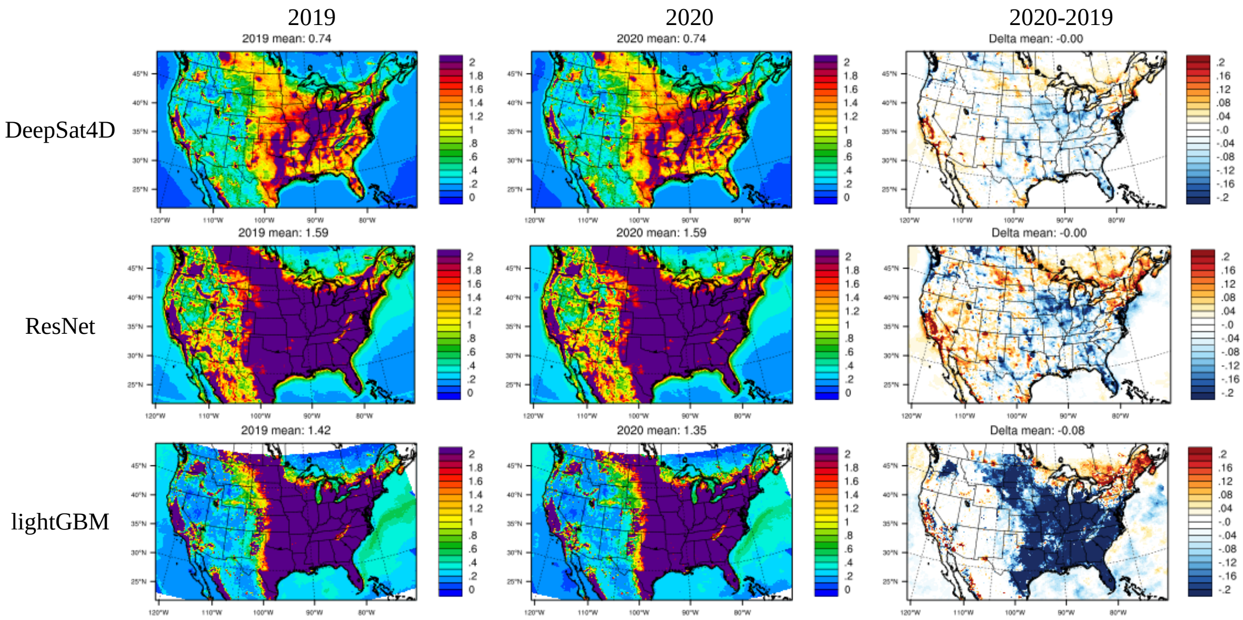

Figure S12. Comparison of the fused concentration among different methods

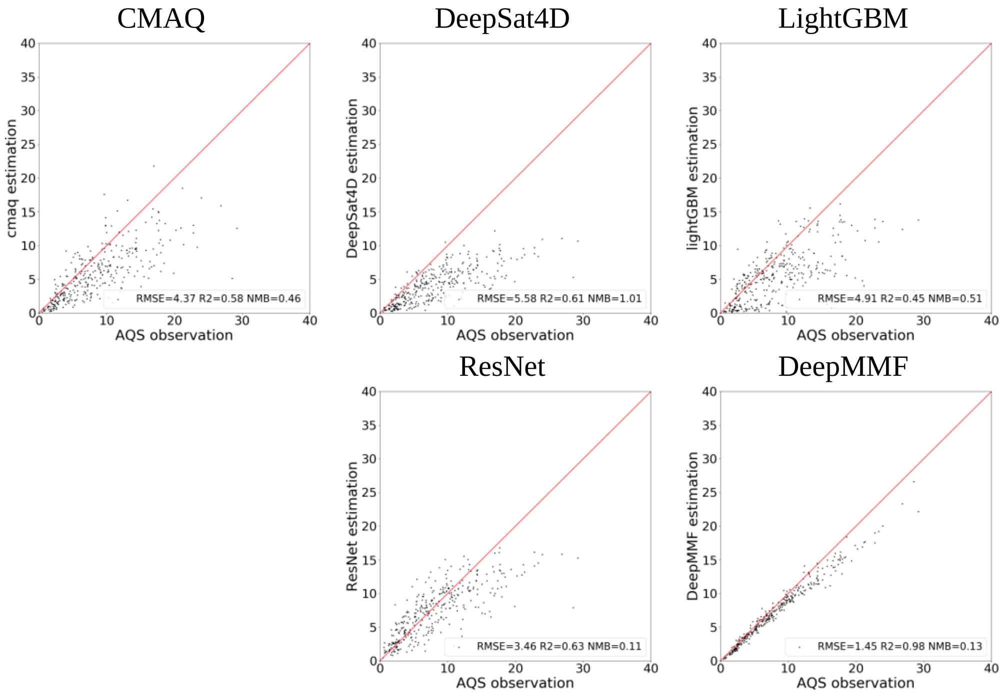

Figure S13. Comparison of estimation surface NO_2 concentration with AQS observations (unit: ppb, annual mean in 2019)

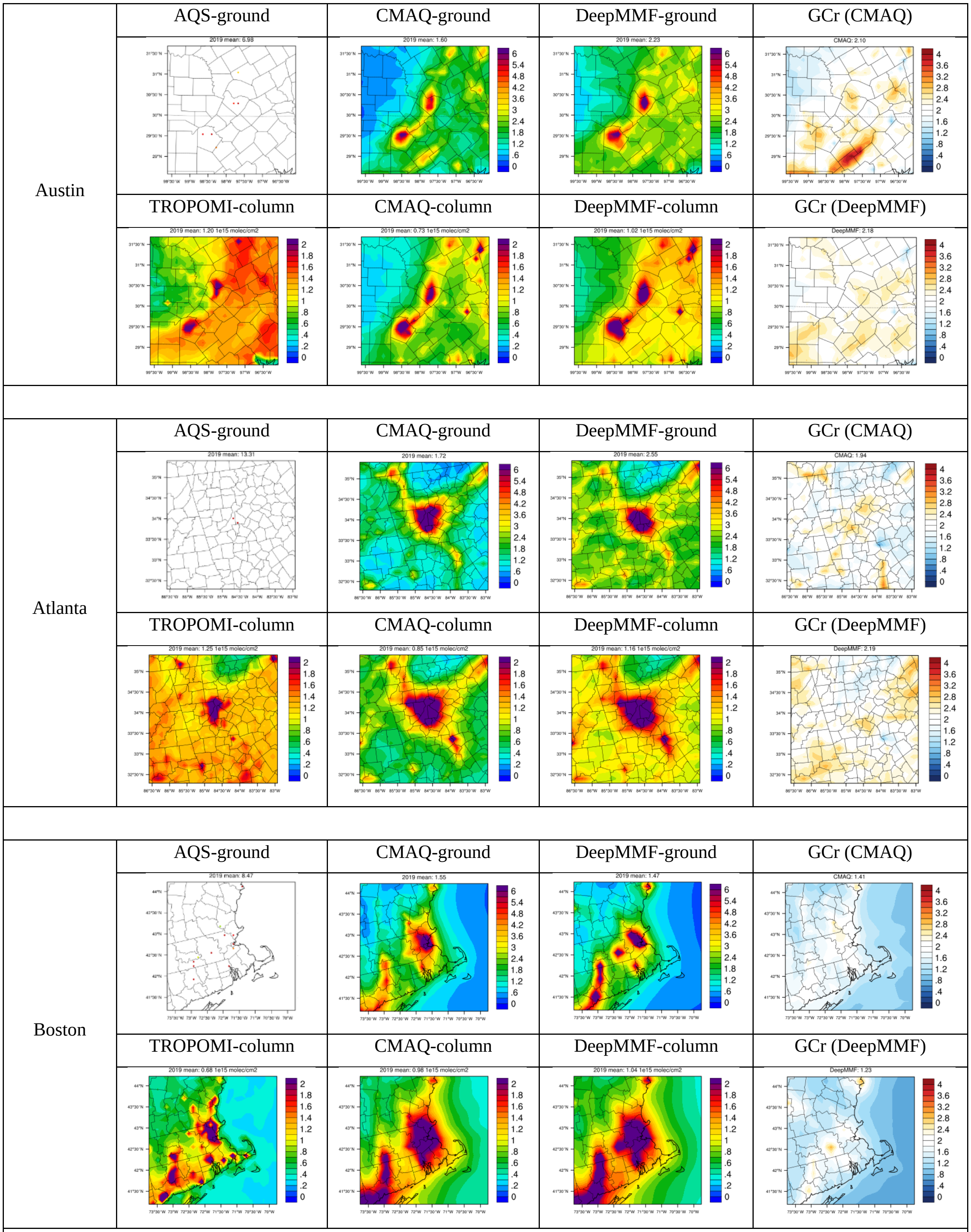

Figure S14. Comparison of simulated, observed, and fused ground and column density of NO_2 and its ground-to-column ratio (GCr) across major US Cities (1/6)

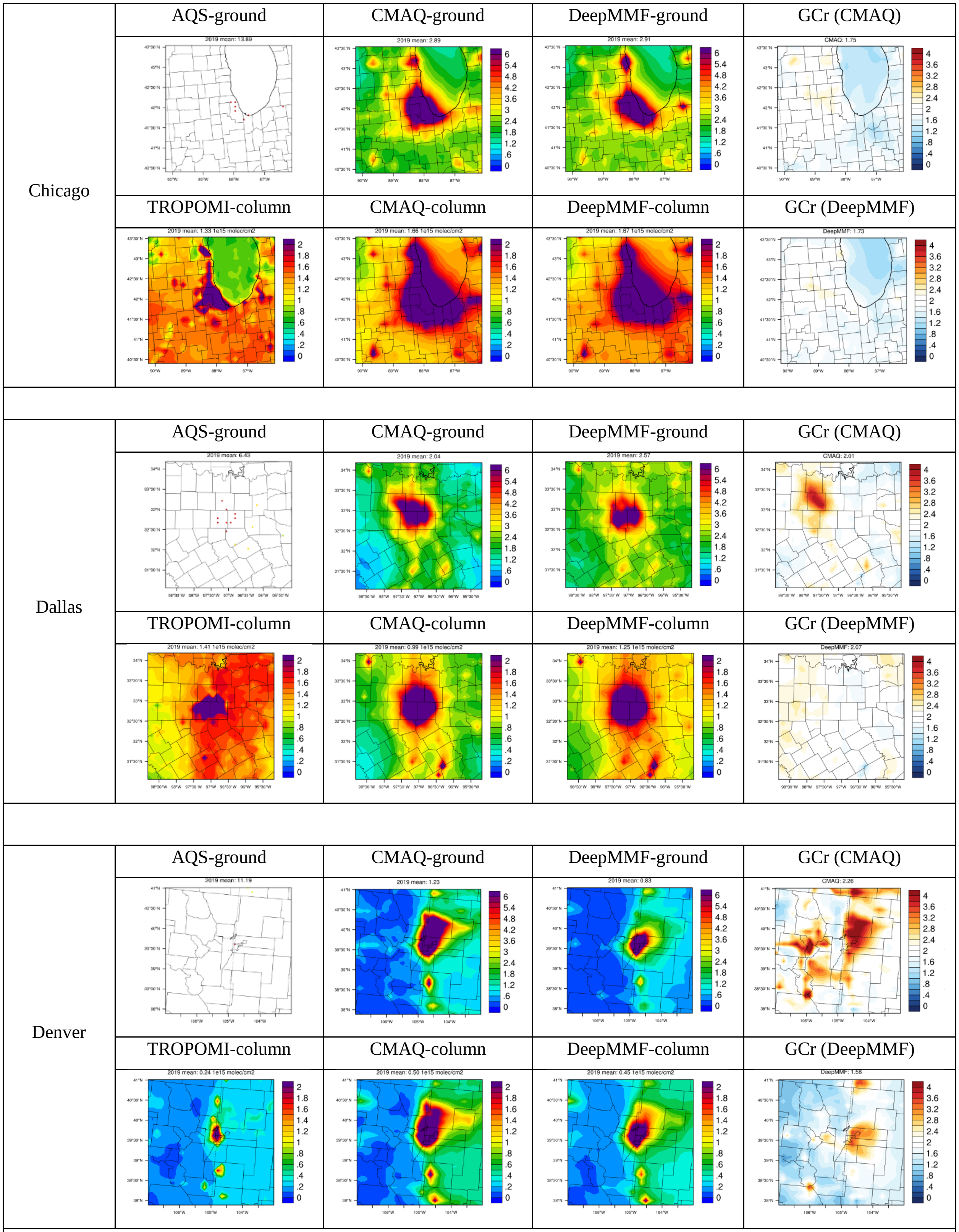

Figure S14. Comparison of simulated, observed, and fused ground and column density of NO_2 and its ground-to-column ratio (GCr) across major US Cities (2/6)

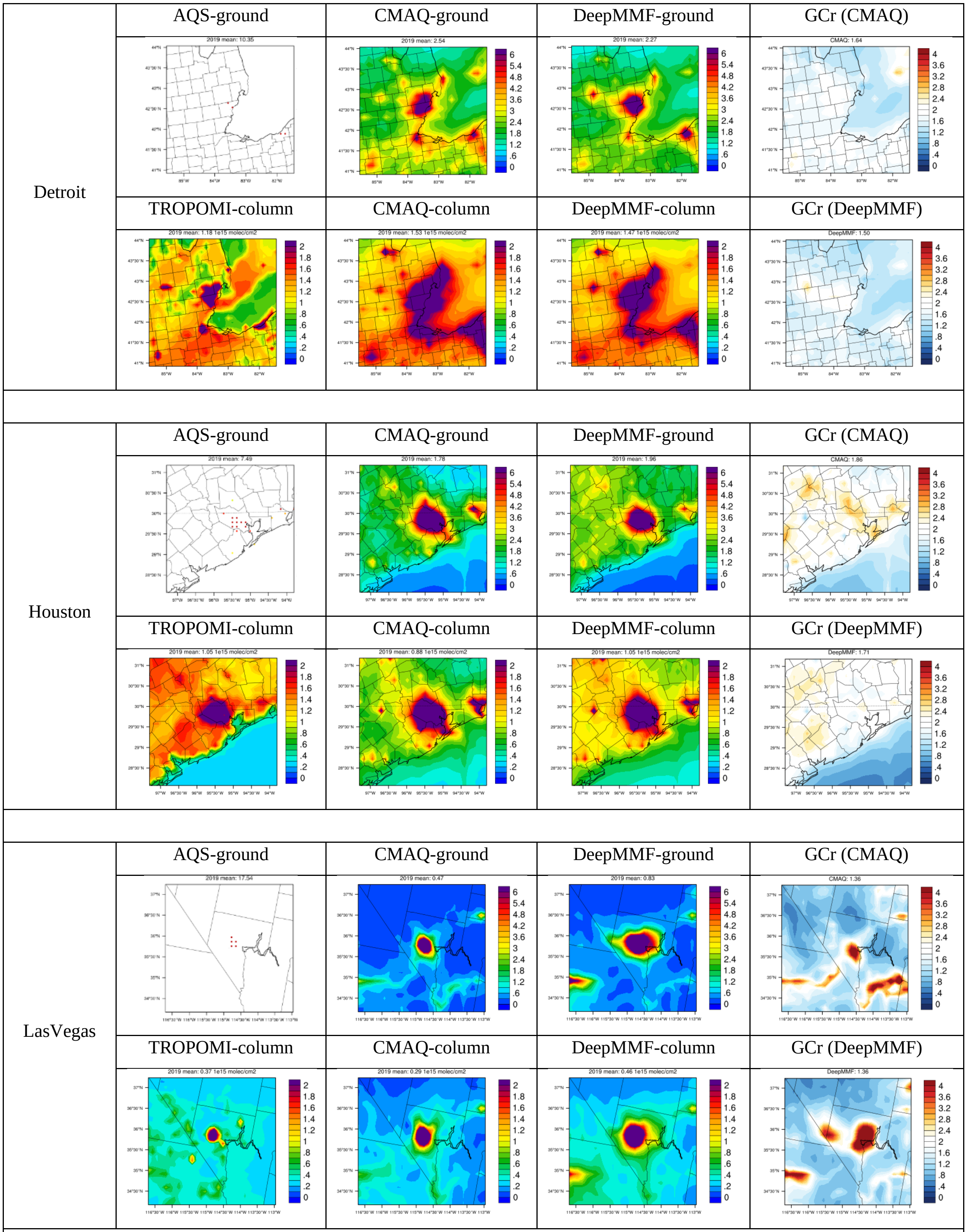

Figure S14. Comparison of simulated, observed, and fused ground and column density of NO_2 and its ground-to-column ratio (GCr) across major US Cities (2/3)

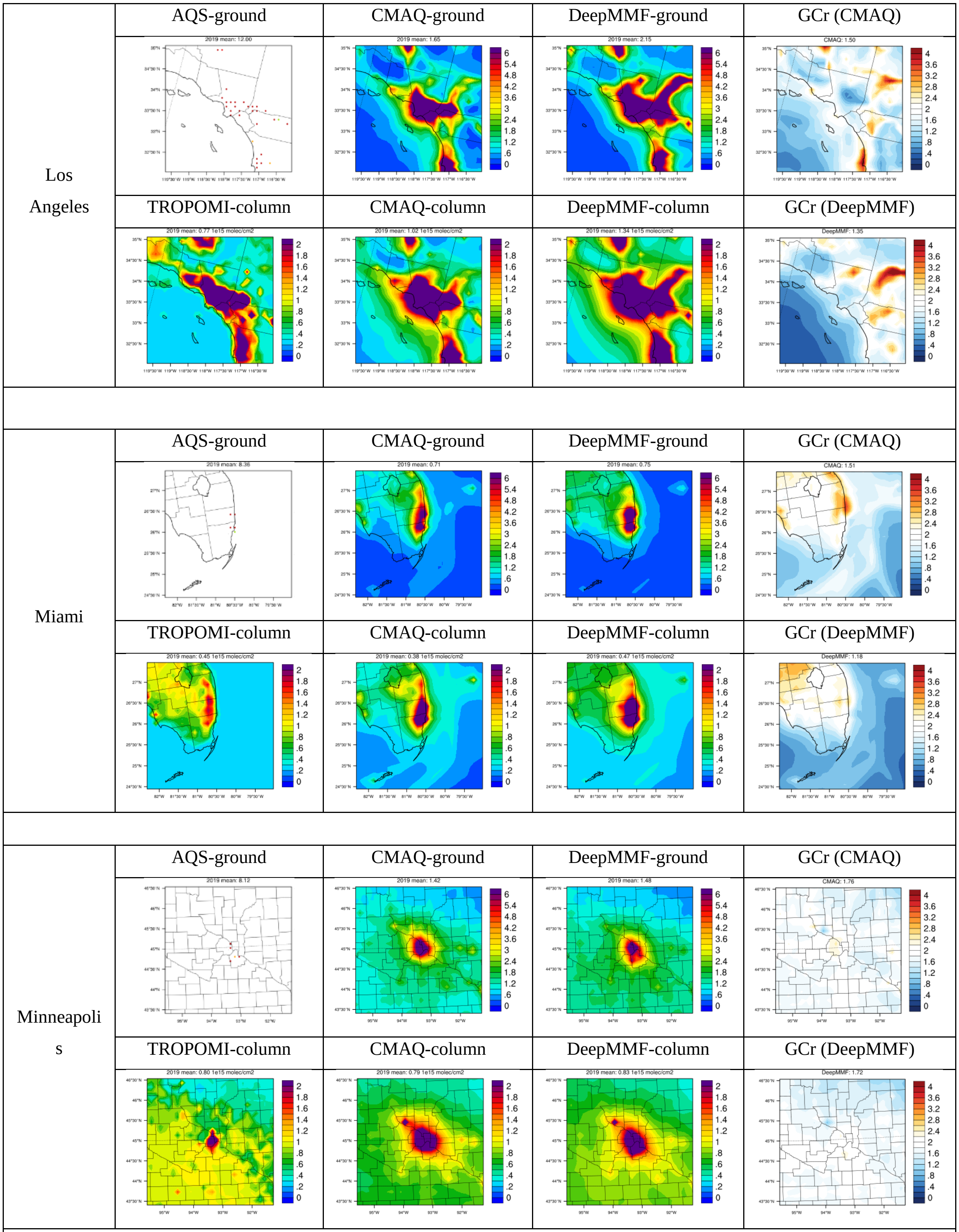

Figure S14. Comparison of simulated, observed, and fused ground and column density of NO_2 and its ground-to-column ratio (GCr) across major US Cities (4/6)

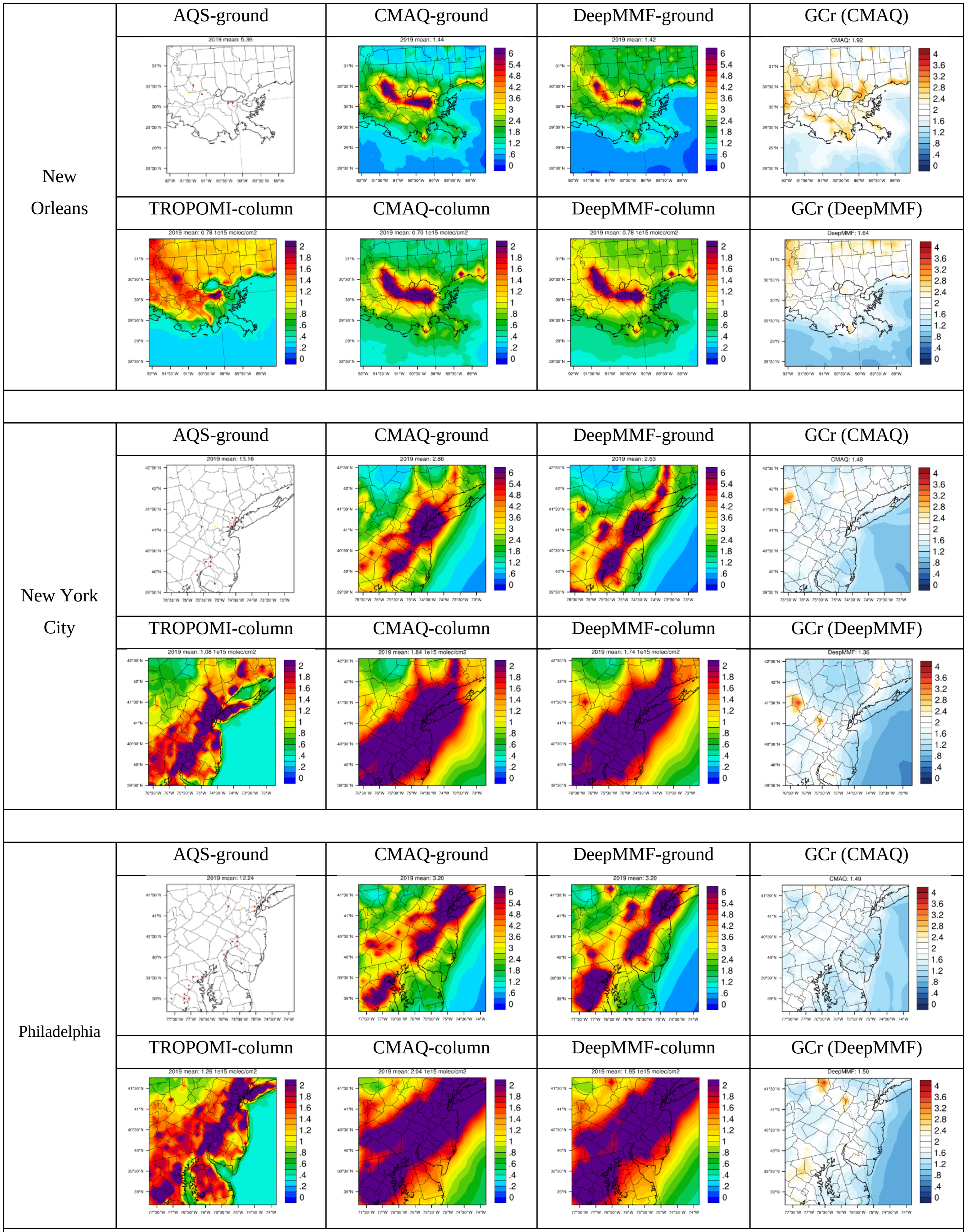

Figure S14. Comparison of simulated, observed, and fused ground and column density of NO_2 and its ground-to-column ratio (GCr) across major US Cities (5/6)

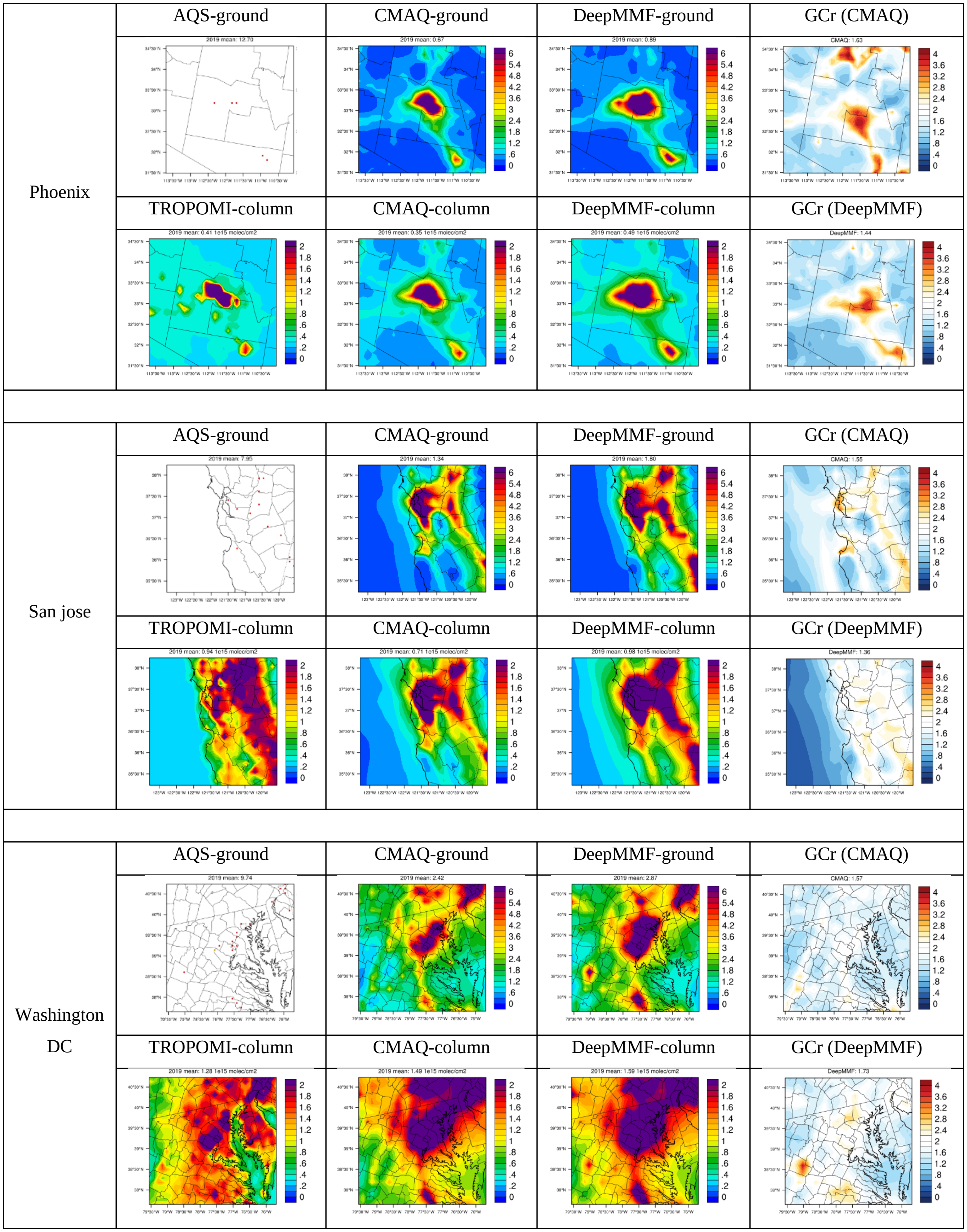

Figure S14. Comparison of simulated, observed, and fused ground and column density of NO_2 and its ground-to-column ratio (GCr) across major US Cities
